# Enhancement of SMN protein levels in a mouse model of spinal muscular atrophy using novel drug-like compounds

**DOI:** 10.1002/emmm.201202305

**Published:** 2013-06-05

**Authors:** Jonathan J Cherry, Erkan Y Osman, Matthew C Evans, Sungwoon Choi, Xuechao Xing, Gregory D Cuny, Marcie A Glicksman, Christian L Lorson, Elliot J Androphy

**Affiliations:** 1 Department of Medicine, University of Massachusetts Medical SchoolWorcester, MA, USA; 2 Derpartment of Dermatology, Indiana University School of MedicineIndianapolis, IN, USA; 3 Department of Veterinary Pathobiology, Bond Life Sciences Center, University of MissouriColumbia, MO, USA; 4 Department of Molecular Microbiology and Immunology, University of Missouri School of MedicineColumbia, MO, USA; 5 Laboratory for Drug Discovery in Neurodegeneration, Harvard NeuroDiscovery Center, Brigham and Women's Hospital and Harvard Medical SchoolCambridge, MA, USA

**Keywords:** drug discovery, SMA, SMN, SMN2, spinal muscular atrophy

## Abstract

Spinal muscular atrophy (SMA) is a neurodegenerative disease that causes progressive muscle weakness, which primarily targets proximal muscles. About 95% of SMA cases are caused by the loss of both copies of the *SMN1* gene. *SMN2* is a nearly identical copy of *SMN1*, which expresses much less functional SMN protein. *SMN2* is unable to fully compensate for the loss of *SMN1* in motor neurons but does provide an excellent target for therapeutic intervention. Increased expression of functional full-length SMN protein from the endogenous *SMN2* gene should lessen disease severity. We have developed and implemented a new high-throughput screening assay to identify small molecules that increase the expression of full-length SMN from a *SMN2* reporter gene. Here, we characterize two novel compounds that increased SMN protein levels in both reporter cells and SMA fibroblasts and show that one increases lifespan, motor function, and SMN protein levels in a severe mouse model of SMA.

## INTRODUCTION

Spinal muscular atrophy (SMA) is a neurodegenerative disorder that presents as progressive muscle wasting and loss of motor function. It is caused by the degeneration of motor neurons, specifically the anterior horn cells of the spinal cord and is one of the leading heritable causes of infant mortality worldwide (Crawford & Pardo, 1996; McAndrew et al, 1997; Pearn, 1978). SMA is caused by a deficiency of the SMN protein. There are two nearly identical SMN genes, the telomeric *SMN1* and the centromeric *SMN2* (Boda et al, 2004; Echaniz-Laguna et al, 1999; Monani et al, 1999). While the protein coding capacity of *SMN2* is identical to that of *SMN1* (Jablonka et al, 2000a), there is a translationally silent nucleotide variation in exon 7 of *SMN2* (Lorson et al, 1999; Monani et al, 1999). This C to T transition results in alternative splicing of *SMN2* and exclusion of exon 7. From *SMN1*, >95% of the transcripts include exon 7 and express the full-length SMN protein. From the *SMN2* mRNA, ∼85% of the messages lack exon 7 (Gavrilov et al, 1998; Gennarelli et al, 1995; Lorson et al, 1999; Monani et al, 1999) and express a truncated form of the protein (SMNΔ7). The SMNΔ7 protein is inactive and cannot fully compensate for the loss of *SMN1* (Burnett et al, 2009; Lorson & Androphy, 2000; Lorson et al, 1998).

*SMN2* is a potent disease modifier for SMA, and there is an inverse relationship between the number of copies of *SMN2* and clinical severity. Most cases of SMA harbour homozygous deletions of the *SMN1* gene but retain at least one copy of *SMN2* (Brahe et al, 1996; Campbell et al, 1997; Hahnen et al, [Bibr b37],[Bibr b38],[Bibr b39]; Jablonka & Sendtner, 2003; Melki, 1997; Talbot et al, 1997; van der Steege et al, 1996; Velasco et al, 1996). The relationship between *SMN2* copy number and disease severity has been confirmed in SMA mouse models (Hsieh-Li et al, 2000; Michaud et al, 2010; Monani et al, 2000). Homozygous deletion of the single copy of the mouse *Smn* gene is embryonic lethal (Schrank et al, 1997). Introduction of two copies of the human *SMN2* transgene supports viability but these animals have motor function defects and an average life span of 4–6 days. Increasing the number of *SMN2* copies decreases disease severity and increases life span. High copy number *SMN2* transgenic mice were phenotypically ‘normal’ (Monani et al, 2000).

Because SMA carriers with only one copy of *SMN1* are clinically asymptomatic, 50% of normal SMN levels should protect from disease. If *SMN2* can be stimulated to express more full length SMN mRNAs, synthesis would be directed towards increased amounts of the active SMN protein (Cherry & Androphy, 2012). Although the threshold level of SMN necessary to maintain motor neurons is not known, only 10–15% of *SMN2* transcripts contain exon 7 and express functional SMN, so doubling or tripling the amount of full length *SMN2* mRNA should be clinically significant (Meyer et al, 2009).

There is no treatment for SMA. Therapeutic modalities for treatment of SMA that are being actively pursued include oligonucleotides to restore *SMN2* exon 7 inclusion, gene transfer using viral vectors, and cell replacement with motor neuron differentiated stem cells (Corti et al, [Bibr b23],[Bibr b24]; DiDonato et al, 2003; Dominguez et al, 2011; Foust et al, 2010; Hua et al, [Bibr b45],[Bibr b46]; Passini et al, [Bibr b74],[Bibr b75]; Porensky et al, 2012; Valori et al, 2010; Williams et al, 2009). Several laboratories have undertaken screens for drug-like compounds that increase cellular levels of the SMN protein from the *SMN2* gene. Compounds that have been shown to increase *SMN2* expression include various histone deacetylase (HDAC) inhibitors, aclarubicin, indoprofen, splicing modifiers, a DcpS inhibitor, anti-terminators, proteasome inhibitors and inhibitors of multiple signalling pathways (Andreassi et al, 2001; Avila et al, 2007; Bowerman et al, [Bibr b7],[Bibr b8]; Burnett et al, 2009; Chen et al, 2012; Farooq et al, 2009; Garbes et al, 2009; Hahnen et al, 2006; Hastings et al, 2009; Heier & DiDonato, 2009; Jarecki et al, 2005; Kernochan et al, 2005; Kwon et al, 2011; Lunn et al, 2004; Makhortova et al, 2011; Narver et al, 2008; Singh et al, 2008; Wolstencroft et al, 2005; Zhang et al, [Bibr b97],[Bibr b98]). Because many of these activators are non-specific and can have off-target effects, their long-term safety remains to be determined. Compounds that have advanced into clinical trials have demonstrated mixed results. There is clearly need for additional drug candidates (Darras & Kang, 2007; Sproule & Kaufmann, 2010; Sumner, 2006).

We previously reported the development of an *SMN2-luciferase* reporter assay to identify compounds that increase SMN expression from the *SMN2* gene (Cherry et al, 2012). This assay has been used at two screening centres to screen over 300,000 compounds (Cherry et al, 2012; Xiao et al, 2011). From a screen of 115,000 compounds at the Laboratory for Drug Discovery in Neurodegeneration (LDDN), 462 hits were identified and 19 ‘high’ priority compounds were selected on the basis of their activity, potency, specificity, lack of overtly toxic functional groups, and potential tractability for chemical modification. Here we report the selection and further characterization of two compounds as potential leads for new SMA therapies.

## RESULTS

### Hit confirmation

As described previously (Cherry et al, 2012), 492 hits were identified from a library of 115,000 compounds. The activity of these compounds was confirmed in the *SMN2-luciferase* cell line and counter-screened with a control cell line expressing luciferase from the minimal SV-40 promoter (*SV40min-luciferase*). Two-hundred and ninety four compounds reproduced >60% increase in luciferase activity and of these 18 were selected on the basis of potency, strength of activation, dose dependency, specificity against luciferase control, and favourable chemical properties. Each of these compounds showed greater than 100% increase in luciferase activity in the *SMN2-luciferase* cell lines and stimulated the control reporter cell lines by less than 40%. All lacked overtly toxic functional groups and had chemical scaffolds that were tractable to chemical modification.

**Figure 1:**
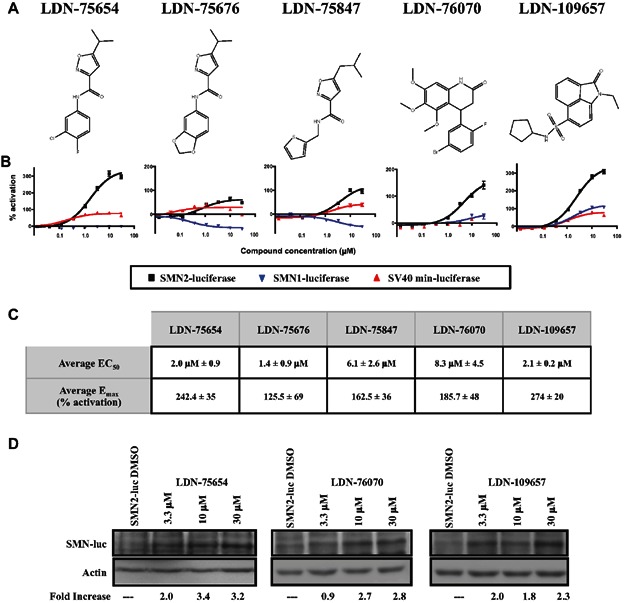
Hit confirmation in the reporter cells Structures of the hits LDN-75654, LDN-75676, LDN-75847, LDN-76070 and LDN-109657.Dose response experiments. Each compound was tested with the reporter cell lines at the concentrations; 0.04, 0.12, 0.37, 1.11, 3.33, 10, 30 µM. Black—*SMN2-luciferase* cells; blue—*SMN1-luciferase* cells; red—*SV40min-luciferase* control cells. *Y*-axis represents % activation over DMSO control. All points were tested in quadruplicate and plotted as mean ± SEM. Curves were created by linear regression using Prism4 (GraphPad Software Inc.).Summary of dose response experiments denoting average potency as EC_50_ (concentration required to achieve 50% of the maximal drug response) and average *E*_max_ (maximal % increase in luciferase activity observed with treatment). For each calculation, *n* ≥ 4 and data are presented as mean ± SEM.Increase in SMN-luciferase fusion protein with hits. *SMN2-luciferase* reporter cells were incubated in presence of increasing amounts of compound for 24 h. Lysates were blotted with antibodies to luciferase and b-actin. Experiments were performed three times. Blots shown are representative.

These compounds were re-screened using the *SMN1-luciferase*, *SMN2-luciferase* cell lines and the *SV40min-luciferase* control cell lines (Supporting Information Fig S1). This panel of reporters allows for the discrimination between compounds that target general SMN transcription and protein expression and turnover and SMN2 specific splicing or other SMN2 specific mechanism, while also allowing for the detection of non-specific inhibitors. Of the 18 compounds re-tested in dose response experiments, all increased luciferase expression by >60% (data not shown). We previously reported preliminary data for 3 of the 18 primary hits from this screen; LDN-72939, LDN-79199 and LDN-109657 (Cherry et al, 2012). Here we further characterize LDN-109657 and two new structural classes (Fig 1A).

The related analogs, LDN-75654, LDN-75676 and LDN-75847 represent a new class of SMN2 inducing compounds. Each contains an oxazole carboxamide backbone. Of these three compounds, LDN-75654 was more potent and had greater activity, with a >240% (3.4-fold increase) in luciferase activity and an average EC_50_ of 2 µM (Fig 1B). LDN-75654 was chosen for further characterization as the preferred lead from this series. LDN-76070 was identified as a singleton in our initial screen. It elicited >180% or 2.8-fold increase in luciferase activity with an average EC_50_ of 8.3 µM (Fig 1B). The dose response curve from LDN-109657 is presented to illustrate the activity and specificity profile, which is similar to that of LDN-76070, but is more potent and promotes higher levels of activation (Fig 1B). The relative activity and potency for these compounds is summarized in Fig 1C.

To confirm that the increased luciferase activity corresponded to an increase in the amount of SMN-luciferase fusion protein, Western blots on lysates from compound treated *SMN2-luciferase* reporter cells were performed with an antibody to luciferase. The level of SMN-luciferase protein increased following incubation of *SMN2-luciferase* reporter cells for 24 h with compounds LDN-75654, LDN-76070 and LDN-109657 (Fig 1D). These increases in protein level were comparable to the increases observed for luciferase activity in the dose response curves (Fig 1B).

### Analysis of mRNA in *SMN2-luciferase* reporter by qRT-PCR

Based on the *SMN2* reporter design, we predict that the compounds identified through this screen could increase SMN protein levels by stimulation of SMN transcription, exon 7 inclusion in the *SMN2* pre-mRNA, or inhibition of SMN protein turnover (Supporting Information Fig S1; Cherry et al, 2012). Comparison of the activity of these compounds in the reporter cell lines can provide insight into the mechanisms of action for these compounds. The *SMN1-luciferase* and *SMN2-luciferase* reporters allow expression of an SMN-luciferase fusion protein from either the *SMN1* or *SMN2* 3.4 kB promoter in the context of their respective cDNA and the genomic exon 6–8 splicing cassette. Activation of the *SMN1-luciferase* reporter would suggest generalized increase in SMN expression or protein levels. Activation of the *SV40min-luciferase* reporter, which drives luciferase expression from the SV40 promoter, would suggest non-specific activity. All compounds presented in Fig 1 displayed some low level of non-specific activation of the *SV40min-luciferase* reporter; none activated *SV40min-luciferase* to the same extent as the activation observed in the *SMN2-luciferase* reporter cell line. For example LDN-76070 and LDN-109657 caused an increase in *SMN2*-*luciferase* levels, but also increased luciferase expression in the *SMN1-luciferase* and *SV40min-luciferase* reporters to a much lesser degree (Fig 1B). These results suggest that these compounds act through a general mechanism that target both *SMN1* and *SMN2*. LDN-75654 and its analogs, LDN-75676 and LDN-75847, increased *SMN2-luciferase* expression but appeared to inhibit luciferase expression from the *SMN1-luciferase* reporter, suggesting that these compounds have target specificity for SMN2 (Fig 1B).

**Figure 2:**
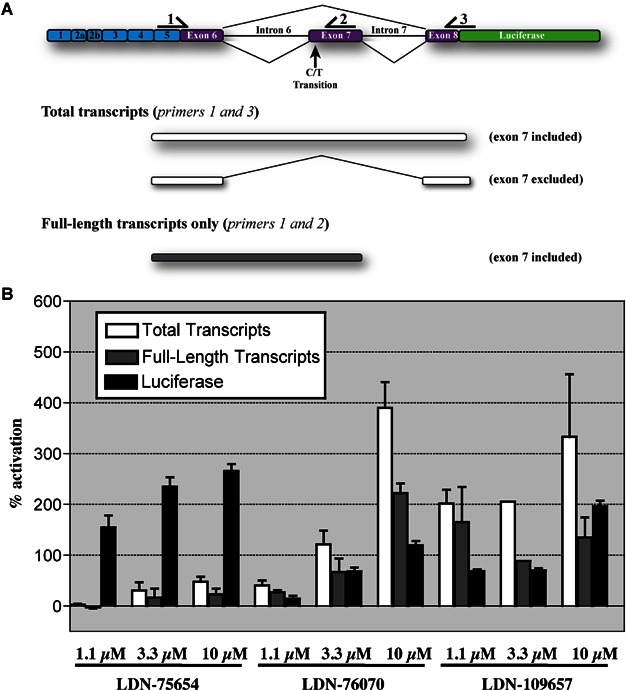
Analysis of SMN-luciferase fusion transcripts by qRT-PCR A schematic of the primer design for qRT-PCR. Primer pairs were chosen to amplify only the SMN-luciferase fusion transcripts but not endogenous SMN. Primer 1 overlaps a unique *Xho* I site. Primers 1 and 2 can only amplify full-length *SMN-luc* transcripts that contain exon 7. Primers 1 and 3 amplify *SMN-luc* reporter transcripts (both exon 7 included and excluded).Graph of qRT-PCR results. Quantitation of luciferase activity (black bars), total reporter transcripts (white bars), and exon 7 included reporter transcripts (grey bars). Transcripts were quantitated by qRT-PCR. Cells were treated at three increasing concentrations for 24 h. Percent increase was calculated in relation to treatment with DMSO and normalized to GAPDH. For each data point, *n* ≥ 3 and data are presented as mean ± SEM.

Quantitative reverse transcriptase PCR (qRT-PCR) was used to analyse *SMN-luciferase* mRNA from control and compound-treated *SMN2*-*luciferase* cells. The upstream primer for each pair overlaps a unique restriction site present only in the reporter and will amplify only the transcripts derived from *SMN-luciferase* reporter (Fig 2A). To measure changes in the level of total *SMN-luciferase* mRNA, primers that amplify both the full-length (exon 7 included) and Δ7 (exon 7 excluded) *SMN-luciferase* transcripts were used (Fig 2A; primers 1 and 3). To specifically measure the amount of full-length exon 7 included *SMN-luciferase* transcripts, a primer in exon 7 was paired with the upstream primer (Fig 2A; primers 1 and 2). For each sample the percent change in the amount of total *SMN-luciferase* transcripts (white bar), full-length exon 7 included *SMN-luciferase* transcripts (grey bar), and *SMN2-luciferase* activity (black bar) was plotted (Fig 2B). Compounds that solely stimulate transcription should predominantly increase the amount of total *SMN-luciferase* transcripts with a proportional increase of exon 7 included transcripts. Compounds that stimulate exon 7 inclusion should increase the amount of full-length exon 7 included transcripts with little to no change in the expression of total *SMN-luciferase* transcripts.

LDN-76070 treatment resulted in an increase in total transcript, with a concurrent increase in exon 7 included mRNA. As would be expected, the increase in SMN-luciferase activity closely mirrored the increase in the exon 7 included transcripts (Fig 2B; grey and black bars). A similar pattern of mRNA expression was observed with LDN-109657. It can be inferred that these compounds increase SMN transcription.

LDN-75654 had a different qRT-PCR profile (Fig 2B). Little to no change in mRNA levels was observed except at its highest concentration. However, the luciferase activity showed a concentration dependent increase, suggesting that LDN-75654 exerts its effect through a post-transcriptional mode-of-action.

### Increases in total SMN protein levels and SMN gems in SMA derived fibroblasts

**Figure 3:**
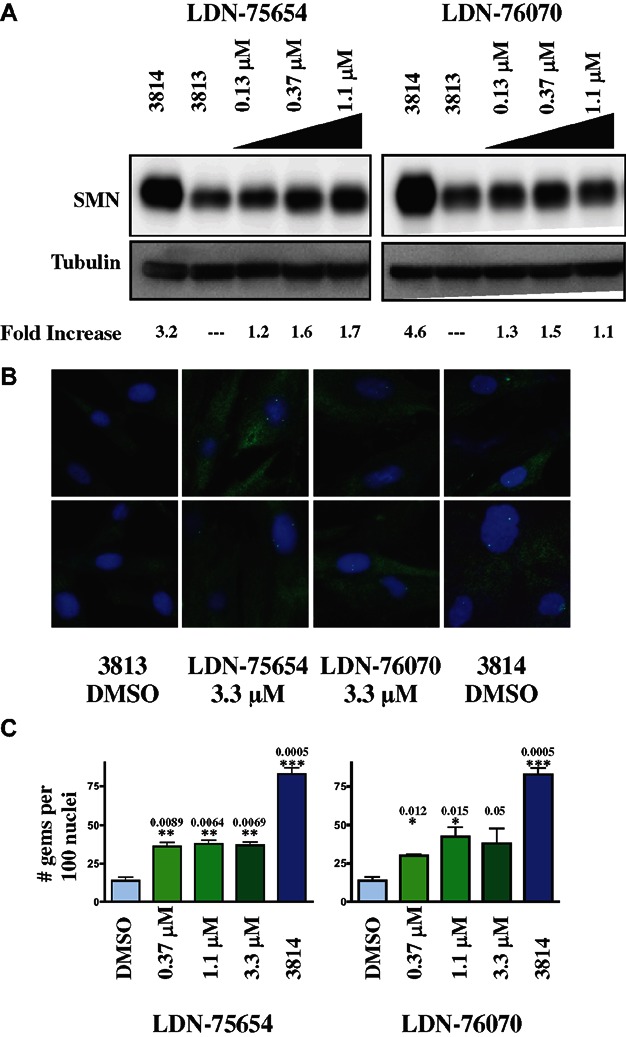
Activity of primary hits in primary human fibroblasts Total endogenous SMN protein in 3813 fibroblasts. Primary human fibroblast lysates from carrier (3814; *SMN1*^+/−^; SMN2^+/+^) and SMA (3813; *SMN1*^−/−^; SMN2^+/+^) derived cells were blotted with antibodies to SMN and α-tubulin. Cells were treated for 48 h with increasing concentrations of compound. Experiments were performed three times.Representative pairs of images of SMN gems in 3813 or 3814 SMA fibroblasts. 3813 primary fibroblast were treated with DMSO, 3.3 µM LDN-76070, or 3.3 µM LDN-75654 for 48 h. (SMN-green and DAPI-blue) The scale bar is 10 µM.Quantification of gem data. Increases in gem numbers were quantified by incubating cells for 48 h in increasing amounts of compound, fixed, and imaged as above. Graphs plot the number of gems per 100 nuclei where 3813 cells are designated by green bars and 3814 by blue bars. Data are presented as mean ± SEM for three experiments and was analysed using Prism4 (GraphPad Software Inc.). Each data point was compared to DMSO control using unpaired *t*-test with *p*-values shown. *p < 0.05; **p < 0.01; ***p < 0.001.

To confirm the ability of the compounds to increase SMN expression from the *SMN2* gene, their effects on endogenous SMN protein levels were examined. It is common practice to use SMA derived primary fibroblasts to assess SMN protein levels in response to SMN2 inducing compounds. We use the 3813 (*SMN1*^−/−^; *SMN2*^+/+^) and 3814 (*SMN1*^+/−^; *SMN2*^+/+^) cells. 3813 cells express low levels of endogenous SMN protein, while 3814 cells, which carry a single copy of *SMN1*, express 3–5 times more full-length SMN protein (Coovert et al, 1997) (Fig 3A).

The activity of LDN-109657 increases SMN protein levels in 3813 fibroblasts by twofold at a concentration of 370 nM (Cherry et al, 2012). LDN-75654 and LDN-76070 were tested for their effect on total endogenous SMN protein levels. 3813 fibroblasts were treated with varying concentrations of each compound for 72 h and protein was quantified by immunoblot. We observed that these primary fibroblasts were sensitive to lower concentrations of compound than those used in the immortalized HEK293 reporter cell lines. The average increase for each of these compounds was determined from multiple experiments. LDN-75654 treatment resulted in dose dependent increases in SMN levels with a maximum average increase of 1.9 ± 0.5-fold at a dose of 1.1 µM. LDN-76070 was most active at a dose of 370 nM with a maximal increase in SMN protein level of 1.8 ± 0.3-fold averaged over three experiments. The immunoblots presented in Fig 3A are from a single experiment and are representative of the overall pattern of SMN expression observed in multiple experiments.

Another useful characteristic of the SMN protein is its localization to large nuclear bodies called ‘gems’ (Lefebvre et al, 1997; Liu & Dreyfuss, 1996). The relative number of gems per nucleus correlates positively with total SMN protein level and inversely with disease severity in a wide array of patient fibroblasts (Coovert et al, 1997; Patrizi et al, 1999). Unlike the Western blot for total SMN protein, the gem assay scores SMN levels on a cell-by-cell basis and focuses on the more stable gem associated pools of SMN protein. This approach is not impacted by the effects these compounds may have on cell proliferation. This assay allows for shorter treatment times and the use of higher compound concentration as is seen in the gem count experiments in Fig 3B and C.

Fibroblasts (3813) were treated as described above for the quantitative immunoblot assay and assayed for SMN immunofluorescent staining in the nucleus. The 3813 cells had an average of 14 ± 4.7 gems per 100 nuclei and the 3814 cells had an average of 84 ± 4.8 per 100 nuclei. The immunofluorescent images in Fig 3B illustrate the increase in the number of gems observed with the addition of 3.3 µM compound for 48 h. Treatment with either compound increased the number of gems in 3813 fibroblasts by nearly twofold at concentrations between 370 nM and 3.3 µM. (Fig 3C).

### Additive effects of compounds used in combination

Based on the qRT-PCR data, we hypothesized that LDN-76070 affects transcription, while LDN-75654 functions at the post-transcriptional level either by stimulating translation or stabilizing SMN protein. To further explore their activities, the compounds were tested together in pairs and individually with the pan HDAC inhibitor suberoylanilide hydroxamic acid (SAHA) in the *SMN2-luciferase* reporter assay. SAHA has been shown to increase SMN protein expression by increasing transcription of the *SMN* gene and by increasing exon 7 inclusion in *SMN2* transcripts (Hahnen et al, 2006). In this assay, the *SMN2-luciferase* reporter cells were treated at six doses increasing from 124 nM to 30 µM of one compound and combined with either DMSO or a second compound at concentrations of 1.1, 3.3, or 10 µM. All data points were collected in triplicate and six-point dose response curves were generated for each pair.

**Figure 4:**
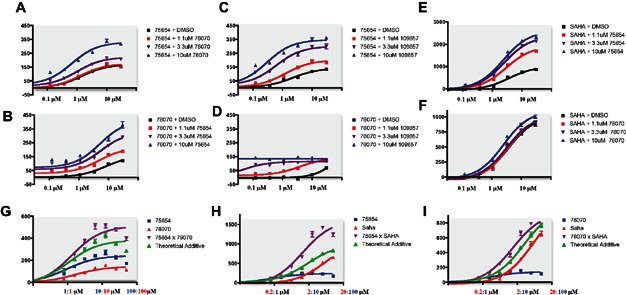
Dose response profiles for compounds in combination **A–F.** Compounds were assayed in a dose response alone or in the presence of 3 fixed concentrations of a second compound (A) LDN-75654 *versus* LDN-76070, (B) LDN-76070 *versus* LDN-75654, (C) LDN-75654 *versus* LDN-109657, (D) LDN-76070 *versus* LDN-109657, (E) SAHA *versus* LDN-75654, (F) SAHA *versus* LDN-76070.**G–I.** For constant ratios combinations, compounds were tested alone or in combination. The combined compounds were tested at concentrations that varied at a constant ratio for all data points. The ratio is based on the difference in EC_50_ values for each compound separately. The blue and red lines illustrate the curves for each compound independently. The green line represents the theoretical additive threshold for the compounds combined. The purple line represents the experimental data for the compounds in combination. (G) LDN-75654 *versus* LDN-76070, (H) SAHA *versus* LDN-75654, (I) SAHA *versus* LDN-76070. Graphs plot the increase in luciferase activity in relation to DMSO treated control. *Y*-axis represents % activation over DMSO. All points were tested in triplicate and plotted as mean ± SEM. Curves were created by linear regression using Prism4 (GraphPad Software Inc.).

When a fixed dose of LDN-76070 was mixed with LDN-75654 in a dose response experiment, the amplitude of *SMN2-luciferase* activation was enhanced in comparison to LDN-75654 alone (DMSO-black line, Fig 4A). This augmentation was observed as the concentration of LDN-76070 increased from 1.1 µM (red), 3.3 µM (purple), and 10 µM (blue). In the reciprocal experiment, a dose response of LDN-76070 in combination with a fixed dose of LDN-75654 revealed similar additive effect (Fig 4B). qRT-PCR analysis demonstrated that LDN-109657 increased the amount total *SMN2-luciferase* transcripts, suggesting that it also acts at the transcriptional level (Fig 2B). As expected, when LDN-109657 was paired with LDN-75654, an additive increase in activity was observed (Fig 4C). However when LDN-76070 was combined with LDN-109657, there was no increase in activity (Fig 4D). Instead, at higher doses, LDN-109657 masked the dose response curve of LDN-76070 eliciting the maximal luciferase irrespective of LDN-76070 concentration. We propose that the increase in response amplitude results from a combination of compounds that cooperate through separate mechanisms or pathways, while an apparent increase in potency with no response in amplitude is the result of additive effects of two compounds with similar modes of action. Pairing SAHA with LDN-75654 produced a dramatic effect, resulting in a >threefold additive increase in activity at all concentrations (Fig 4E). Pairing SAHA with the putative transcriptional activator LDN-76070 resulted in only a slight additional increase in activation at the highest concentrations of SAHA (Fig 4F).

To further characterize the relationship between the two scaffolds, we examined them in combination using a constant ratio design (Chou, 2006). In this experiment, each compound is tested alone or in combination with a second compound. In each case, the compounds are paired in a constant ratio that is centered on their EC_50_. For these experiments, the effective EC_50_ value for both LDN-75654 and LDN-76070 was 6.25 µM while the EC_50_ for SAHA was 1.25 µM. The constant ratio between LDN-75654 and LDN-76070 was 1:1 and the constant ratio between these compounds and SAHA was 5:1. The activity of each compound individually (red and blue) at each data point can be added to the activity of its partner for that same data point and a plot for the theoretical additive curve is used as a threshold for additivity (Fig 4G–I; green line). If the experimental curve (purple) for the compounds in combination matches this curve, the effect of these compounds in combination is additive. If the experimental curve is above the theoretical additive curve, the activities of these compounds are more than additive and may be acting synergistically. If the compounds fall below the theoretical curve, they may be acting through a similar or related mechanism and thus diminishing the activity of each compound individually. It is also possible that such compounds are acting in opposition to one another and the interaction could be considered antagonistic. While the single dose curves showed increased amplitude with combinations of LND-76070 with 75654 (Fig 4A and B) and SAHA with LDN-75654 (Fig 4E), the constant ratio design suggests that the relationships between these compounds are synergistic (Fig 4G and H). In this set of experiments, the combination of LDN-76070 with SAHA also resulted in luciferase activity that was greater than additive (Fig 4I). However, the additive effects were less than those observed in the pairing of LND-76070 with 75654 and SAHA with LDN-75654 (Fig 4G and H).

### Efficacy in SMNΔ7 SMA mice

LDN-75654 and LDN-76070 were examined for aqueous solubility, mouse liver microsome stability, and HDAC inhibitor activity. Due to its limited solubility and poor chemical tractability, LDN-109657 was excluded from further analysis. These data are summarized in Table 1. LDN-75654 was soluble at a maximum concentration of 10 µg/ml in PBS (pH 7.0) and had a relatively short half-life in mouse live microsomes. LDN-76070 appeared to be more stable and had a 40 min half-life in the mouse liver microsomes, however, its maximum solubility in PBS was <10 µg/ml.

**Table 1:** Preliminary lead characterization

LDN-75654	LDN-76070	
242.4 ± 35	185.7 ± 48	Activity luc reporter (% activation)
2.0 µM ± 0.9	8.3 µM ± 4.5	Potency luc reporter (EC_50_)
15 min	40 min	Stability in mouse liver microsomes
10 µg/ml	<10 µg/ml	Solubility assay (µg/ml in PBS)
–	–	HDAC inhibitor activity
n.d.	++++ (*n* = 16)	Ability to right in Δ7 mice^a^
no increase	>3-fold	Protein increase in Δ7 mice^b^
n.d.	10 days	Increase in median survival in Δ7 mice^c^

n.d., not determined; a, ability to right is determined in time to right assay; b, protein increase is determined in spinal cord and brain in relation to DMSO treated SMA Δ7 littermates; c, median survival for DMSO treated SMA pups was 6 days.

To evaluate these compounds in animals, pilot experiments were initiated using SMNΔ7 (Smn^−/−^, SMN2^+/+^, SMNΔ7^+/+^) (Le et al, 2005). The compounds were administered either by intraperitoneal (i.p.) or intracerebroventricular (i.c.v.) injection into the SMA mice as previously described (Baughan et al, 2006; Coady et al, 2008). Animals were treated for 3 days, starting on postnatal day 1 (PND). On PND3, mice were sacrificed and tissues were harvested.

**Figure 5:**
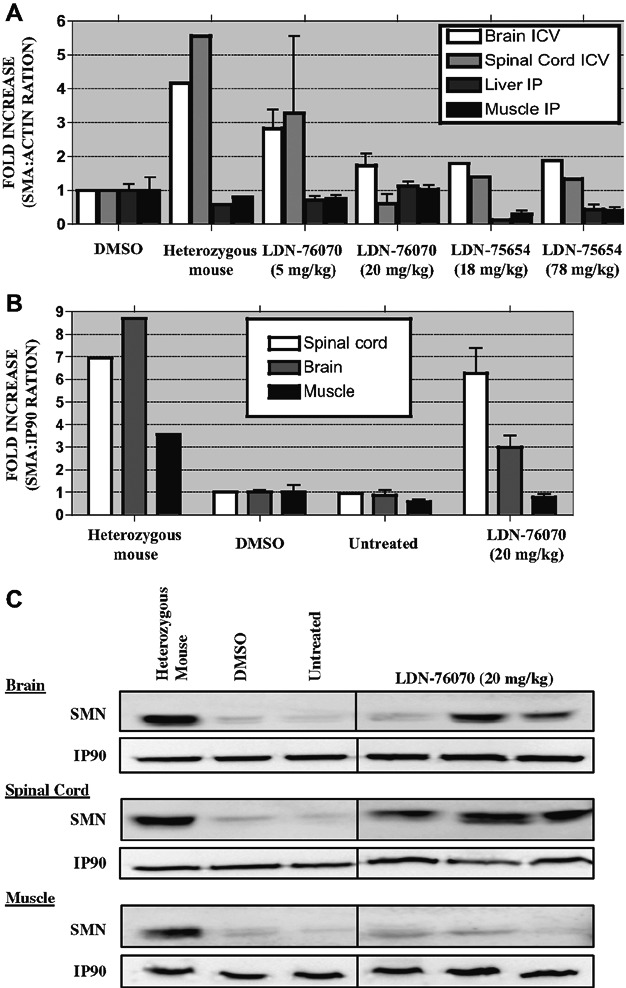
Increase in total SMN protein in SMNΔ7 mice Pilot experiments in SMNΔ7 mouse model. Animals were treated daily for 3 days starting at PND1 with compounds at various concentrations. Tissues were harvested from two sets of animals, brain and spinal cord from animals that received compound by i.c.v. and muscle and liver from animals that were injected by i.p. SMN protein measured by semi-quantitative immunoblot and normalized to actin. Increases in protein are presented as fold increase when compared to DMO treated SMA neonates.Quantification of SMN protein levels in LDN-76070 treated SMNΔ7 animals. Starting on PND2, Animals were injected daily with 20 mg/kg of compounds by i.p. On PND7, animals were sacrificed and tissues from brain, spinal cord, and muscle were harvested. SMN protein measured by semi-quantitative immunoblot and normalized to the IP90 (calnexin) as a loading control. For each data point, *n* = 2 and data are presented as mean ± SEM. Increases in protein are presented as fold increase when compared to DMO treated SMA neonates.Representative immunoblots of total SMN protein expression in the brain, spinal cord, and muscle of untreated, DMSO treated, or LDN-76070 treated SMNΔ7 animals. The images are from the same blot with the same exposure.

Animals treated with 5 mg/kg LDN-76070 showed increased SMN protein in spinal cord and brain (Fig 5A). Treatment with 20 mg/kg LDN-76070 caused a lesser increase in SMN protein in both the spinal cord and brain while promoting a slight increase in SMN levels in the liver. Treatment with LDN-75654 had no effect on SMN protein levels in either the muscle or liver. In the brain and spinal cord, LDN-75654 induced a slight increase in SMN protein (Fig 5A). However, this increase was inconsistent and varied among the small number of treated animals. A second, appropriately powered study was set up to confirm these data. Due to the weak and inconsistent responses observed with LDN-75654 in the pilot experiments, LDN-76070 was chosen as the primary lead for *in vivo* validation in the SMA mouse model. We chose to confirm *in vivo* efficacy using a dose of 20 mg/kg to achieve higher *in vivo* concentrations following i.p. administration.

LDN-76070 was administered to SMNΔ7 mice once daily by i.p. starting on PND 2. Tissues were harvested from three treated animals on PND 7 and compared to tissues from asymptomatic heterozygous littermates, untreated, and DMSO (vehicle) treated animals. Brain, spinal cord and muscle were assayed for total SMN protein with the house keeping gene IP90 (calnexin) as a loading control (Fig 5C). The mean increase of SMN protein levels in the spinal cord of LDN-76070 mice was over sixfold greater than untreated or DMSO treated animals. This was close to 90% of the protein detected in the heterozygous littermates (Fig 5B). SMN protein levels also increased in brain by over threefold, which corresponded to >30% of SMN detected in the brains of heterozygous animals. There was no increase in the amount of SMN protein in the muscle with LDN-76070 treatment.

**Figure 6:**
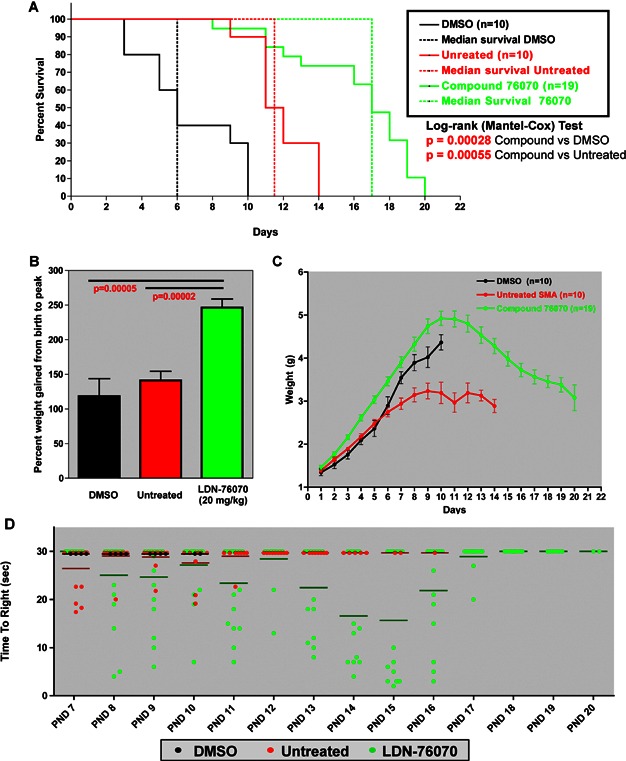
Activity of LDN-76070 in SMNΔ7 mice Animals were injected with 20 mg/kg of each compound LDN-76070 (green; *n* = 19) and compared to untreated (red; *n* = 10) or DMSO treated animals (black; *n* = 10) Compound was administered i.p. once daily starting on post natal day 2 (PND). Kaplan–Meier curve illustrates a significant increase in lifespan with compounds (Log-rank (Mantel–Cox) test; *p* = 0.00028 in comparison to DMSO and *p* = 0.00055 in comparison to untreated animals).Average percent weight gained from peak to birth. LDN-76070 treated animals (green) show a significant increase in percent weight gained from birth to peak in comparison to DMSO (black) and untreated animals (red)(*p* = 0.00005 and 0.00002, respectively; unpaired *t*-test).The average weight for surviving members of each cohort is plotted daily. Data are presented as mean ± SEM.Treated animals (green) displayed an increase in the ability to tight themselves when compared to untreated (red) or DMSO treated animals (black).

To assess the impact of LDN-76070 on survival, daily treatment of SMNΔ7 animals with 20 mg/kg was continued as long as feasible. The lifespan of treated animals was compared to that of untreated and DMSO treated animals. We observed a decrease in life span with DMSO treatment. The median survival of untreated animals was 11.5 days, while DMSO treated animals had a median survival of 6 days. Treatment with LDN-76070 increased median survival to 17 days. This corresponded to a 180% (2.8-fold) increase in lifespan over DMSO control animals and 48% (1.4-fold) over untreated animals (Fig 6A). In both comparisons, the increase in lifespan was statistically significant with, *p* = 0.00028 and *p* = 0.00055, respectively.

Another metric to gauge the potential efficacy of new therapies in the SMNΔ7 mice is weight gain from birth to peak. This measurement is different than total weight as it incorporates the variability in birth weight for this very severe model. Typically mice range in birth weight from 1.2 to 1.9 g. The average birth weight for the SMNΔ7 mice was 1.42 ± 0.2 g. In the survival experiments, DMSO and untreated animals reached an average peak weight of 2.93 ± 1.5 and 3.33 ± 1.1 g, respectively. The weights of the animals were recorded each day and the average weight gain from birth to peak for all animals in each tested group was calculated (Supporting Information Fig S2). LDN-76070 treated mice produced a ∼250% gain in peak weight, while weight increases in the DMSO treated and untreated animals were less than 150% (Fig 6B). The average number of days required to reach peak weight varied from 6.5 in DMSO, to 9.1 in untreated, and to 10.4 days in LDN-76070 treated animals.

We also calculated the daily average weights for each of the surviving animals, including the sicker, smaller animals that died prematurely (on or before day 6). Since the median survival of the DMSO treated animals was 6 days, the apparent increase in their average weight on days 7 through 10 was the result of the limited number of animals remaining; each of these had slightly higher weights (Fig 6C and Supporting Information Fig S2). However, the animals treated with LDN-76070 still reached a higher average peak weight of 5.05 ± 0.9 g and had substantially longer life span compared to either of the control groups (Fig 6C and Supporting Information Fig S2).

The ‘time to right’ (TTR) test is a measure of gross motor function (Butchbach et al, 2007b). All DMSO treated and four of ten untreated SMA pups failed this test, the remaining animals displayed an intermittent ability to right themselves. Furthermore, none of the control animals showed the ability to right after PND 10 (DMSO group) and PND11 (Untreated group). SMA pups treated with LDN-76070 displayed a dramatic improvement in the ability to turn over. Twelve of nineteen LDN-76070 treated animals displayed improved motor function over the course of multiple days. This was most dramatic at PND 14 and 15 when the average righting time for the surviving population was under 20 s. A few treated pups were able to turn over in as few as 5 s and maintained at this level of gross motor function up to PND 16 (Fig 6D). Collectively, the data show statistically significant extension of life and motor capabilities with LDN-76070 treatment in comparison to DMSO and untreated animals.

## DISCUSSION

SMA is primarily caused by the loss or mutation of both copies of the *SMN1* gene. The *SMN* gene is required for viability (Schrank et al, 1997) and the copy number of *SMN2* inversely correlates to disease severity (Feldkotter et al, 2002; Harada et al, 2002; Monani et al, 2000; Wirth et al, 1999). The potential to express functional full-length SMN protein and the presence of at least one copy in nearly all SMA patients makes *SMN2* an attractive therapeutic target for the treatment for SMA. Although the threshold level of SMN protein necessary to maintain motor neurons has not been adequately determined, it is reasonable to predict that doubling or tripling the amount of full length SMN protein should be sufficient to prevent or diminish clinical progression. Motor neurons appear intact at birth in SMA murine models, so there appears to be a temporal window for restoration of SMN and survival of motor neurons (McGovern et al, 2008). Consistent with this, an inducible mouse model of SMA demonstrated that whole-body restoration of SMN decreases disease severity even if induction occurs after the onset of symptoms (Lutz et al, 2011).

As a potent disease modifier for SMA, *SMN2* has become a high priority target for SMA therapeutics. Many of the compounds in development are either general neuroprotective compounds or compounds re-purposed from other indications. HDAC inhibitors including sodium butyrate, SAHA, phenyl butyrate, trichostatin A (TSA), LBH589 and M344, are the most commonly studied class of SMN inducers and have all been reported to increase SMN transcription (Andreassi et al, 2004; Avila et al, 2007; Brichta et al, 2003; Chang et al, 2001; Garbes et al, 2009; Hahnen et al, 2006; Riessland et al, 2006; Sumner et al, 2003). Sodium butyrate and SAHA have also been shown to increase exon 7 inclusion in *SMN2* transcripts. The HDAC inhibitor VPA has also been characterized as a splicing enhancer for *SMN2* (Brichta et al, 2003).

*SMN*2 based reporters have been used to identify novel compounds for the treatment of SMA. Our original splicing cassette was used in low-throughput mode to identify the phosphatase inhibitor sodium orthovanadate (Zhang et al, 2001), aclarubicin (Andreassi et al, 2001) and indoprofen (Lunn et al, 2004). These compounds have been shown to increase full-length SMN protein expression from the *SMN2* gene. Sodium orthovanadate, a phosphatase inhibitor, might affect SR (serine/arginine rich) protein phosphorylation state and thereby modulate SMN splicing (Zhang et al, 2001). Interestingly, both aclarubicin and sodium orthovanadate activities are enhanced in the presence of the transcription factor Stat5 (signal transducers and activators of transcription 5), while Stat5 knockout abrogates their effect (Ting et al, 2007). The hormone prolactin can activate Stat5, which in turn increases SMN expression (Farooq et al, 2011). In the same study, prolactin treatment in the SMNΔ7 mice improved gross motor function and increased survival. This suggests that SMN expression may be regulated by a signal transduction pathway in response to cytokines or growth factors. Indoprofen, a non-steroidal anti-inflammatory drug, was also identified using our first generation reporter assay. The mechanism of action for indoprofen has not been determined but recent evidence suggests that it has anti-terminator activity. Aminoglycosides also have anti-terminator activity and have been shown to stabilize the SMNΔ7 protein and presumably increase its functionality (Heier & DiDonato, 2009; Mattis et al, 2008). An independent *SMN2* promoter screen was used to identify the 2,4 diaminoquinazoline series of compounds (Jarecki et al, 2005). The quinazoline compounds may act by binding to and inhibiting the scavenger decapping enzyme, DcpS (Singh et al, 2008). Other modes of action being explored include proteasome inhibition, SMN stabilization, inhibition or activation of signal transduction, and targeted regulation of exon 7 inclusion and are currently at different stages of development.

We previously reported a new screen that combined the benefits of the splicing and transcriptional assays for SMN2 expression (Cherry et al, 2012). In that report, we described three novel compounds that increased SMN expression in the reporter cells and were confirmed in 3813 SMA derived fibroblasts. Early medicinal chemistry efforts revealed that these compounds lacked the characteristics desired for further development. Here we characterize two new scaffolds, LDN-75654 and LDN-76070, which induce expression of full-length SMN from *SMN2*. Both compounds increase SMN-luciferase expression in the reporter assay and levels of endogenous SMN protein in 3813 SMA derived primary fibroblasts with low micromolar EC_50_s. Neither of the compounds displayed inhibition of HDAC 3, 6 or 8 activity *in vitro*. These compounds also displayed selectivity for *SMN2-luciferase* expression when tested in two additional cell lines for specificity, *SMN1-luciferase* and the *SV40min-luciferase*. These compounds have been tested in other high throughput screens without being identified as hits, suggesting the utility of the multi-faceted screening platform.

RT-PCR analysis confirmed that LDN-76070 and LDN-109657 increased the amount of total SMN-luciferase fusion transcripts, with a concomitant increase in the amount of full-length transcripts (Fig 2B). LDN-76070 does not share structural similarity with other compounds shown to increase SMN expression. The pathway and targets involved in its activity are still unknown. LDN-75654 also increased the amount of detectable SMN-luciferase fusion protein, but it promoted little change at the mRNA level. This suggests that LDN-75654 functions post-transcriptionally, perhaps by increasing translation efficiency or decreasing SMN protein turnover. Post-transcriptional regulation of SMN expression is not without precedent. The aminoglycosides are anti-terminators that act post-transcriptionally by allowing read through of the stop codon in exon 8 of the SMNΔ7 transcript (Mattis et al, [Bibr b62],[Bibr b64]; Wolstencroft et al, 2005). The additional amino acids produced by this read-through stabilize the SMNΔ7 protein product. Due to the design of our screen, activity of anti-terminators like the aminoglycosides would not be detected. LDN-75654 is not structurally similar to aminoglycosides or other compounds reported to regulate SMN expression. However, the oxazole carboxamide backbone of LDN-75654 is similar to that of leflunomide, a pyrimidine synthesis inhibitor used to treat rheumatoid arthritis (Bartlett et al, 1991; Ruckemann et al, 1998). Leflunomide was also active in our reporter screen and was identified independently using our *SMN2-luciferase* reporter cells (PubChem CID = 3899). We are currently examining the nature of this similarity.

Investigation of the combinatorial effects of these scaffolds could provide further insight into their general mechanism of action and might enhance their therapeutic efficacy. Combining LDN-75654 with the transcriptional activators LND-76070, LDN-109657, or SAHA produced a greater than additive stimulation of the *SMN2-luciferase* reporter. This effect was most apparent when LDN-75654 was combined with SAHA, which has been shown to increase both SMN transcription and exon 7 inclusion. We propose that the complementation seen with these compounds is a confirmation that the compounds are working through separate and distinct mechanisms to induce *SMN2* expression. The effect of LDN-76070 was partially masked by the addition of SAHA. SAHA appears to overwhelm the transcriptional machinery and blunt the efficacy of LDN-76070, suggesting that these compounds stimulate *SMN2-luciferase* expression through similar or overlapping pathways. The observation that these two compounds still produce a greater than additive effect in the constant ratio experiments may be due to the secondary effect that SAHA has on the splicing efficiency and inclusion of exon 7. The dramatic increase in *SMN2-luciferase* activation observed with the combination of SAHA with LDN-75654 suggests that LDN-75654 augments SMN expression in addition to the splicing and transcriptional increases produced by SAHA, possibly through effects on protein stability or translation.

Systematic delivery of LDN-76070 promoted greater than threefold increases in SMN protein levels in the brain and spinal cord, suggesting the ability to penetrate the blood brain barrier. It dramatically increased lifespan and gross motor function in these animals as evidenced by the improvements in TTR tests. LDN-76070 displayed excellent stability in mouse liver microsomes, half-life of 40 min, but has low aqueous solubility. While LDN-75654 promoted increases in SMN expression in both the reporter assay and SMA derived fibroblasts, treatments with this compound in pilot experiments with the SMNΔ7 mice resulted in weak and inconsistent increases in SMN protein levels. LDN-75654 had a relatively short half-life of 15 min in *in vitro* mouse liver microsome assays. We assume that this lack of metabolic stability accounts for its poor activity in the animals.

There has been an unprecedented increase in the therapeutic pipeline for SMA (Cherry & Androphy, 2012; Lorson & Lorson, 2012). Unlike early studies focused on neuroprotective agents including carnitine, riluzole and more recently olesoxmine and ceftriaxone, the compounds describe in this manuscript are designed to target SMN2 expression. The largest class of compounds that is currently being studied for their ability to increase SMN expression is the HDAC inhibitors. These have shown modest extension of lifespan in SMA model mice (Chang et al, 2001; Riessland et al, 2010), however, VPA and phenylbutyrate failed to exhibit significant clinical efficacy in human SMA protocols. Other drug-like small molecules that have increased lifespan of SMA mice include an orally bioavailable quinazoline analog, the aminoglycoside TC007, and prolactin (Butchbach et al, 2010; Farooq et al, 2011; Mattis et al, 2009).

LDN-76070 induces *SMN2* transcription, while LDN-75654 acts post-transcriptionally. Neither inhibited HDAC activity *in vitro*. LDN-76070 treated animals lived an average 11 days longer than DMSO treated littermates, representing an increase of lifespan of more than 180%. Despite the apparent DMSO toxicity, LDN-76070 treatment still increased the lifespan of these animals by 48% over that of untreated animals. The chemical scaffolds described here, derived from a high-throughput screen of a chemical diversity library to increase SMN protein, show promise as these compounds exhibited the predicted activities in mice. Further work is necessary to improve their pharmacological properties and determine their precise mode of action.

## MATERIALS AND METHODS

### Cell culture

Cells were incubated at 37°C with 5% CO_2_. HEK-293 cells were grown in D-MEM (Gibco 11995) with 10% foetal bovine serum (FBS Atlas) and 1× pen-strep (Gibco 15140). Reporter cell lines containing *SMN1*, *SMN2* or control luciferase reporter were selected and maintained in D-MEM with 10% FBS and 1× pen-strep with 200 µg/ml hygromycin B (Invitrogen 10687-010). Primary human fibroblasts were grown in D-MEM with 10% FBS and 1× pen-strep.

### Luciferase assay

*SMN2-luciferase*, *SMN1-luciferase*, and *SV40min-luciferase* reporter cells (Cherry et al, 2012) were plated in D-MEM in the absence of hygromycin B and allowed to adhere 24 h prior to addition of compound at 37°C with 5% CO_2_. 25,000 cells were added per well to 96 well white tissue culture treated plates. The final DMSO concentration in each well was 0.1%. Plates were incubated for 25 h. Firefly and renilla luciferase expression were measured using Dual Glo luciferase substrate (Promega E2920) on the Envision (Perkin–Elmer). Raw data were collected as counts per second (CPS) with an integration time of 0.1 s. For normalization all raw data points were transformed from CPS to percent activation over basal expression in relation to the DMSO treated control wells.

For the constant ratio combination studies, compounds are then assayed singly or in pairs at concentrations that equal 8× EC_50_, 4× EC_50_, 2× EC_50_, EC_50_, 0.5 EC_50_, 0.25 EC_50_ and 0.125 EC_50_. For example, when LDN-75654 and SAHA were combined, LDN-75654 was assayed alone at 50, 25, 12.5, 6.25, 3.1, 1.6 and 0.78 µM; SAHA was tested alone at 10, 5, 2.5, 1.25, 0.62, 0.31 and 0.16 µM; and they were combined in a ratio of 5:1 with paired concentrations of 50:10, 25:5, 12.5:2.5, 6.25:1.25, 3.1:0.62, 1.60:0.31, and 0.78:0.16 µM.

### SMN protein detection

For analysis of SMN-luciferase fusion, cells were treated with compound or DMSO for 25 h. Cells were lysed with 100 mM Tris pH 8.0, 100 mM NaCl, 0.1% NP-40, 8.0 M Urea, and protease inhibitor. Each sample was separated on a 10% SDS–PAGE gel, transferred to Immobilon-P membrane (Millipore IVPH00010) and blotted for the SMN-luciferase fusion with anti-luciferase antibody (Promega, G7541) as well as actin (Sigma A2066) or α-tubulin (DM1a; Sigma T6199).

For detection of SMN protein in patient fibroblasts, 8,000 cells per cm^2^ were plated 24 h prior to drug addition. Fresh media and compound were added every 24 h. After 72 h, cells were harvested, washed with cold PBS, and lysed as above. We have determined that 10 µg total protein per lane is within the linear range for immunoblot detection of SMN and α-tubulin. Western blots were probed for SMN with the 4f11 mouse monoclonal antibody and α-tubulin.

Quantification of protein was performed with Fujifilm LAS-4000 Multifunctional Imaging System. The signal intensity was measured for each band on an immunoblot, normalized to the loading control, and the fold increase was determined in relation to the appropriate DMSO treated control.

### Microscopy and gem analysis

Gem counts were performed as previously described (Mattis et al, 2006). Briefly, cells were grown on poly(D) lysine treated cover slips, fixed in methanol, and stained with DAPI and reacted with a SMN monoclonal antibody and an Alexa-488 conjugated secondary antibody. Approximately 100 cells from were visually scored for the number of gems and the number of gems per 100 nuclei calculated.

**The paper explained**

PROBLEM:

SMA is the most common cause of infant mortality worldwide. SMA has a carrier rate of 1 in 40 and the disease incidence is 1 in 11,000 live births. It is a neurodegenerative disorder that can progress rapidly. In the most severe cases, symptoms present within the first 6 months of life and death usually occurs within the first 2 years. There is no approved treatment for SMA. New oligo and gene replacement based therapies are showing promise. Existing small molecule therapies have not been effective in human clinical trials.

RESULT:

The presence of the *SMN2* gene in nearly all SMA patients provides an attractive therapeutic target for the treatment of SMA. We present two new series of small molecules that increase the levels of SMN protein derived from the *SMN2* gene. The compounds in these series utilize separate and potentially unique mechanisms of action. They are active in SMN reporter cell lines, primary SMN patient fibroblasts, and, in the case of compound LDN-76070, display *in vivo* efficacy with the SMAΔ7 mice.

IMPACT:

There is a clear need for new therapeutic small molecules in the SMA drug pipeline. The data presented here confirms that these new compounds can increase SMN protein levels in both cells and an SMA model animal. The exciting *in vivo* proof-of-principle data with LDN-76070 in the SMNΔ7 mice confirms that it can cross the blood brain barrier in neonates and induce protein levels in the spinal cord to nearly 90% percent of that found in asymptomatic littermates that are heterozygous for *Smn1*. LDN-76070 also promotes an increase in survival and improvement in gross motor function in these animals. Medicinal chemistry efforts are underway on compounds in both series to improve their pharmacokinetic characteristics; including solubility, metabolic stability, and potency. These compound represent two new and unique series of compounds that might be developed as clinical leads for the treatment of SMA.

### PCR and RT-PCR

For analysis of SMN-luciferase fusion expression, compounds were tested at three concentrations that display maximal activity in the luciferase assay. Cells were treated as described above for the luciferase assay. Cells were harvested by trypsinization, neutralized with trypsin inhibitor, and washed. 10% of each cell pellet was resuspended and plated into three wells of a 96-well dish and used to analyse luciferase activity by DualGlo luciferase assay (Promega E2920). RNA was isolated from the remaining cells using Trizol Reagent (Invitrogen 15596-026). cDNA was generated using the Improm-II Reverse Transcription System (Promega A3801).

The forward primer pair recognizes the exon 5–6 junction, which includes a restriction site that was engineered into the reporter and will exclude amplification of endogenous *SMN* mRNA. The reverse primers recognize either exon 7 or luciferase for detection of full-length or total SMN-luciferase transcripts respectively. For a reference control, we amplified cDNA from the housekeeping gene glyceraldehyde-3-phosphate dehydrogenase (GAPDH).

Primer sequences were as follows; SMN exon5-FWR (5′-catttccttctggaccactcgag-3′), Luciferase-REV (5′-atagcttctgccaaccgaacgg-3′), Exon7-REV (5′-taaggaatgtgagcaccttccttc-3′), GAPDH-REV(G3A) (5′-tccaccaccctgttgctgta-3′), and GAPDH-FWR (G3S) (5′-accacagtccatgccatcac-3′).

qPCR was performed as described in the protocol for iQ SybrGreen Supermix (BioRad 170-8882) using an Eppendorf Mastercycler ep realplex 4 real-time PCR machine. Reactions were incubated for a 10 min 94°C hot start followed by 45 cycles of the following: 94°C for 45 s, 60°C for 15 s, 72°C for 45 s. Melting curves for each reaction were obtained. Each sample was assayed in triplicate and every plate contained a five-point cDNA dilution course to calculate amplification efficiency for each primer pair. The Pfaffl method was used to determine the change in transcript levels relative to the DMSO and normalized to GAPDH (Pfaffl, 2001).

### Animal procedures and experiments

All animal experiments were carried out in accordance with protocols approved by the Animal Care and Use Committee of the University of Missouri. Original breeder pairs of *Smn*^+/−^; *SMN2*^+/+^; SMNΔ7^+/+^ mice were purchased from The Jackson Laboratory (JAX® Mice and Services, 610 Main Street Bar Harbor, ME 04609 USA). Offspring were genotyped on the day of birth as previously described (Coady & Lorson, 2010) using primer sets for the *Smn* gene: mSmn-WT FWD (5′-tctgtgttcgtgcgtggtgacttt-3′) and mSmn-WT REV (5′-cccaccacctaagaaagcctcaat-3′) and for the *Smn* knockout: SMN1-KO FWD (5′-ccaacttaatcgccttgcagcaca-3′) and SMN1-KO REV (5′-aagcgagtggcaacatggaaatcg-3′) utilizing multiplex PCR on tail biopsy material. Both treated and untreated control SMA mice were raised with two unaffected heterozygous siblings to control for litter size. ICV injections were performed as previously described (Coady et al, 2008; Passini & Wolfe, 2001). Briefly, mice were immobilized via cryo-anesthesia and injected using µl calibrated sterilized glass micropipettes. The injection site was approximately 0.25 mm lateral to the sagittal suture and 0.50–0.75 mm rostral to the neonatal coronary suture. The needles were inserted perpendicular to the skull surface using a fibre-optic light (Boyce Scientific Inc.) to aid in illuminating pertinent anatomical structures. Needles were removed after 5 s of discontinuation of plunger movement to prevent backflow. Mice recovered for 5–10 min in a warmed container until movement was restored.

Time-to-right (TTR) was performed on flat surface and the test was terminated at 30 s. If an animal had not turned by this time, it was recorded as ‘Failure.’ TTR success rate and speed tests were initiated on PND7 since unaffected animals start to turn over at this time; trials concluded on PND20.

## For more information

OMIM for Spinal Muscular Atrophy: http://omim.org/entry/253300

Families of Spinal Muscular Atrophy: http://www.fsma.org

FightSMA: http://www.fightsma.org

SMA foundation: http://www.smafoundation.org

Muscular Dystrophy Association: http://www.mda.org

Association Française contre les Myophathies (AFM): http://www.afm-telethon.com/

HarvardNeurodiscovery: http://www.neurodiscovery.harvard.edu/research/lddn_2.html

## Supplementary material

As a service to our authors and readers, this journal provides supporting information supplied by the authors. Such materials are peer reviewed and may be re-organized for online delivery, but are not copy-edited or typeset. Technical support issues arising from supporting information (other than missing files) should be addressed to the authors.
