# Supplementary material for: Enhancement of SMN protein levels in a mouse model of spinal muscular atrophy using novel drug-like compounds
Source: EMBO Mol Med. 2013 Jun 5;5(7):1035–50. doi: 10.1002/emmm.201202305 (PMC3721476; doi:10.1002/emmm.201202305)
Supplement: Supplementary file 1 [file emmm0005-1035-SD1.pdf]

Enhancement of SMN protein levels in a mouse model of spinal muscular atrophy using novel drug-like compounds

Jonathan J. Cherry, Erkan Y. Osman, Matthew C. Evans, Sungwoon Choi, Xuechao Xing, Gregory D. Cuny, Marcie A. Glicksman, Christian L. Lorson, and Elliot J. Androphy

Corresponding author: Jonathan Cherry, Indiana University School of Medicine

Review timeline:

Submission date:	29 November 2012
Editorial Decision:	31 January 2013
Revision received:	14 February 2013
Editorial Decision:	03 May 2013
Revision received:	20 March 2013
Editorial Decision:	25 March 2013
Revision received:	27 March 2013
Accepted:	02 April 2013

Transaction Report:

(Note: With the exception of the correction of typographical or spelling errors that could be a source of ambiguity, letters and reports are not edited. The original formatting of letters and referee reports may not be reflected in this compilation.)

Editor: Roberto Buccione

1st Editorial Decision 31 January 2013

Thank you for the submission of your manuscript to EMBO Molecular Medicine. We are very sorry that it has taken so long to get back to you on your manuscript. In this case we experienced unusual difficulties in securing three willing and appropriate reviewers.

You will see that, while two Reviewers are generally supportive of your work albeit with a number of concerns, Reviewer 2 raises an issue that questions the conclusiveness and actual relevance of the results. Altogether these concerns prevent us from considering publication at this time. I will not dwell into much detail, as the evaluations are self-explanatory. I would like, however, to highlight the main points.

Reviewer 1 suggests a number of important shortcomings in the overall presentation quality and style and suggests a number of remedies. I agree that the manuscript in its current form would need some improvement in terms of readability and accuracy. S/he has also indicated a few other comments and editing suggestions directly in the manuscript file that I am attaching to this letter or sending via separate cover.

Reviewer 2 has one main but critically fundamental concern related to the effects of DMSO toxicity. S/he notes that DMSO alone appears to significantly decrease survival of the SMN delta7 mice and administration of the drug only increases survival to what the normal survival would normally be. A number of options are suggested to remedy this, including DMSO toxicity dose-response trials, verification that the actual SMA electrophysiological phenotype is being corrected and others. I

cannot but stress that this fundamental issue must be thoroughly and convincingly addressed by dispelling any ambiguity on the interpretation and exact significance of the results. Finally, Reviewer 2 would also like to know the rationale for choosing the higher LDN 76070 dosage in the animal models notwithstanding the apparently higher efficacy of the lower dosage.

Reviewer 3 is generally supportive but asks you to carefully discuss the potential advantage of the new compounds described in the manuscript with respect to others and to also consider the issues of DMSO solubility and blood brain barrier penetration with respect to applicability.

While publication of the paper cannot be considered at this stage, we would be prepared to consider a suitably revised submission, with the understanding that the Reviewers' concerns must be fully addressed with additional experimental data where appropriate and that acceptance of the manuscript will entail a second round of review.

Please note that it is EMBO Molecular Medicine policy to allow a single round of revision only and that, therefore, acceptance or rejection of the manuscript will depend on the completeness of your responses included in the next, final version of the manuscript.

As you know, EMBO Molecular Medicine has a "scooping protection" policy, whereby similar findings that are published by others during review or revision are not a criterion for rejection. However, I do ask you to get in touch with us after three months if you have not completed your revision, to update us on the status. Please also contact us as soon as possible if similar work is published elsewhere.

I look forward to seeing a revised form of your manuscript as soon as possible.

***** Reviewer's comments *****

Referee #1 (Comments on Novelty/Model System):

I believe that this article is worthy of publication. There are a profusion of drug discovery for smn2 induction. However most have resulted in a nugatory SMN induction and minimal impact on SMA murine survival This represents one of the most potent small molecule inducers of SMN with the greatest impact on survival yet published. The concept of treating a recessive disorder by inducing what I refer to as a rescuing paralog is an important one in the treatment of genetic disorders

Referee #1 (General Remarks):

Comments:
generally a srong paper although hard to follow in places
They state "Based on the design of the reporter, we hypothesize that the compounds identified through this screen would increase SMN protein levels by regulating overall SMN transcription, exon 7 inclusion in the SMN2 pre-mRNA, or the half-life of the SMN protein (Cherry et al, 2012). "
perhaps a sentence or two describing the assay architecture would be useful.
This reviewer was unable to grasp the import of reporter cell lines, SMN2-luciferase, SMN1-luciferase, and SV40 min-luciferase. hence had difficulty grasping the import of figure 1B. Can these be described more fully, possibly a simple schematic of the assays in supplementary figures.
the authors states "Interestingly, both aclarubicin and sodium orthovanadate activities are enhanced in the presence of the transcription factor Stat5 (signal transducers and activators of transcription 5), while Stat5 knockout abrogates their effect (Ting et al, 2007). This suggests that SMN expression may be regulated by a signal transduction pathway in response to cytokines or growth factors."
this is true, the lab of the late Hung Li showed this as cited and Farooq et al have showed profound SMN upregulation with prolactin activation of stat5, this work might be cited
The authors state that LDN-75654 also increased the amount of detectable SMN-luciferase fusion protein, but it promoted little change at the RNA level. This suggests that LDN-75654 functions post-transcriptionally, perhaps by increasing RNA stability,
This cannot be so as an increase in mRNA stability would increase mRNA levels
they state "18 were selected on the basis of potency, strength of activation, dose dependency, specificity against luciferase control, favorable chemical properties, and commercial availability."

commercial availability doesn't appear to be a factor

the acronym mRNA and RNA are used interchangeably, there should be more consistency use of mRNA
figure 1c define Bmax
figure 1d should say SMN-luc not SMN
the peak weight of the treated mice would be useful to know and should be given
the incidence of 1 in 6000 is too high..the accurate number is 1 in 11,000
Sugarman E.A., Nag an N., Zhu H., Akmaev V.R., Zhou Z., Rohlfs E.M., Flynn K., Hendrickson B.C., Scholl T., Sirko-Osadsa D.A. et al. (2012) Pan-ethnic carrier screening and prenatal diagnosis for spinal muscular atrophy: Clinical laboratory analysis of >72,400 specimens. Eur. J. Hum. Genet., 20, 27-32.
it would be useful but is not essential to have immunohistochemical analysis showing SMN induction in motor neurons.
other comments and editing suggestions are given in the attached word file with tracking changes on

Referee #2 (Comments on Novelty/Model System):

This reviewer has concerns that the mice used in the study (untreated typically live to 15 days), but DSMO toxicity results in aniamls living to median survial of 6.7 days, and the drug proposed brings the survival to 16 days.

Referee #2 (General Remarks):

The manuscript, "Enhancement of SMN protein levels in a mouse model of spinal muscular atrophy using novel drug-like compounds." by Jonathan Cherry, Erkan Osman, Matthew Evans, Sungwoon Choi, Xuechao Xing, Gregory Cuny, Marcie Glicksman, Christian Lorson, and Elliot Androphy describes a screen of compounds to increase SMN levels using a novel reporter system developed by the laboratory. The authors have performed the screen in good detail with meaningful results in the screens, and subsequent in vitro testings for SMN increases.
Several points are confusing:

The authors note: "In animals treated with 5 mg/kg LDN-76070 there was an increase in SMN protein in spinal cord and brain (Fig. 5A). Treatment with 20 mg/kg LDN-76070 caused a lesser increase in SMN protein in both the spinal cord and brain while promoting a slight increase in SMN levels in the liver". It is unclear why the authors then chose to go in vivo with the 20 mg/kg dosage versus the 5 mg/kg which showed better increases.

Concerning is the DMSO toxicity, and then the drug increasing survival to what normal SMA delta 7 animals live to. It is suggested that the authors do not hide this fact, and should put untreated animals on the survival curve as well as it is unclear whether the drug does anything to improve survival versus diminish a DMSO toxicity effect. Has the authors considered treating the dams instead of new born pups? that may be able to circumvent the DMSO toxicity in a new born pup. Also, a lower dose may have better effects (and decreases the DMSO concentration). I would like to see a dose response of DMSO toxicity, and an attempt to capture increase in life span with the drug when animals live a normal lifespan. Another way to address this is to perform electrophysiology in the mice. Are the authors truly correcting the SMA electrophysiological phenotype? There is excitement by this reviewer on the screen and potential here, but in present state, the authors appear to be overly optimistic on the drug candidates chosen with signficant hurdles that are only briefly touched.

Referee #3 (Comments on Novelty/Model System):

My concern is described as major points in the referee comment. The new compound identified in this study has low solubility (requires DMSO), therefore, there are still lot to do to proceed to the clinical application.

I recommend this article can be published as a short report (when appropriately revised), since the study mainly focused to develop new therapeutic compounds for SMA with limited elucidation of disease mechanism.

Referee #3 (General Remarks):

Spinal muscular atrophy (SMA) is a child motor neuron disease, most of which are caused by the homozygous deletion of SMN1 gene. As a result, SMA patients' cells express only SMN2 protein, which is highly homologous to SMN1 but is less functional due to the exclusion of exon 7 of its gene. Increasing SMN2 protein level is considered to be a viable therapeutic target for SMA. Authors have developed a new high-throughput screening assay to identify small molecules that increase the expression of full-length SMN, from a SMN2 reporter gene.
By the use of this assay, they identified and characterized two new compounds that increased SMN protein levels in both reporter cells and SMA fibroblasts. Further, one compound extended lifespan, improved motor function, with increased SMN protein levels in a mouse mode of SMA. The experiments are well designed and the quality of data overall is good. Still there are questions and concerns to be addressed.

Major points:
1. To date, many studies (more than 20 publications) have identified the compounds to increase the level of SMN2 proteins. In light of these, the novelty and the advantage of new compound should be clearly addressed. The impact of prolonged lifespan in this study can be discussed in comparison with other preclinical studies.

2. In the discussion, authors mentioned that the compound LDN-76070 showed low solubility to the water (requires DMSO to solubilize). In addition, the penetrance to the central nervous system (through the brain-blood barrier (BBB)) of this compound may be dependent on the immature BBB of the newborn animal. Still, a substantial effort seems to be required to overcome these problems in order to proceed to the clinical application to these compounds.

Minor points:

1. page 4, line 13: There is no treatment for SMA. Italic font should be corrected.
2. p9, lines 15, 16. Fold changes of SMN increase, 1.9 fold (1.1 uM of LDN-75654), 1.8 fold (370nM of LDN-76070) do not match with the value provided in the figure 3A.
3. p10, line 8. The data provided in Figure 3C does not seem to show that the dose-dependent effect of the compounds on the number of gems.
4. Page 17-19: discussion regarding development of LDN-76070 seems too long. It can be shortened.
5. Figure 3B: Scale bar should be included.
6. Figure 5A, B: Although HET seems heterozygous mice, it should be noted in the legend. LDN-75654 (18mg/kgG): "G" should be eliminated.
7. Figure 5B: what is IP90?

1st Revision - authors' response 14 February 2013

Referee #1 (Comments on Novelty/Model System):

I believe that this article is worthy of publication. There are a profusion of drug discovery for SMN2 induction. However most have resulted in a nugatory SMN induction and minimal impact on SMA murine survival. This represents one of the most potent small molecule inducers of SMN with the greatest impact on survival yet published. The concept of treating a recessive disorder by inducing what I refer to as a rescuing paralog is an important one in the treatment of genetic disorders.

Referee #1 (General Remarks):

1. Requested a description of the reporters used.

This has been addressed in the text and in new Supplemental Figure 1. The detailed description of this assay was recently published and this reference now also appears in the methods section.

2. Requested discussion of the role of prolactin and its role in the regulation of Stat5 and SMN.

This has been addressed, cited, and is now included in the discussion

3. Was concerned with the statement "This suggests that LDN-75654 functions post-transcriptionally, perhaps by increasing RNA stability" This cannot be so as an increase in mRNA stability would increase mRNA levels

This has been corrected in the text.

4. Was concerned with the statement "18 were selected on the basis of potency, strength of activation, dose dependency, specificity against luciferase control, favourable chemical properties, and commercial availability."

This has been corrected in the text.

5. The acronym mRNA and RNA are used interchangeably, there should be more consistency use of mRNA.

This has been corrected in the text.

6. In figure 1c define Bmax.

The correct term is Emax. This denotes the maximal response achievable by a drug. This has been corrected in Figure 1. Emax and EC50 have been defined in the text and figure legend for Fig. 1.

7. Figure 1d should say SMN-luc not SMN.

This has been corrected in the Figure 1d

8. The peak weight of the treated mice would be useful to know and should be given.

This information is now included in a new figure panel 6C and discussed in the text.

9. The incidence of 1 in 6000 is too high…the accurate number is 1 in 11,000.

We have changed this number in the "Paper Explained" narrative.

10. Other comments and editing suggestions are given in the attached word file.

We appreciate and have incorporated the reviewer's suggestions.

11. It would be useful but is not essential to have immunohistochemical analysis showing SMN induction in motor neurons.

It would prove challenging to quantifiably detect changes in SMN protein levels by IHC in these cells. We will plan to examine the NMJ structure in a future report using newer analogues from these series.

Referee #2 (Comments on Novelty/Model System):

This reviewer has concerns that the mice used in the study (untreated typically live to 15 days - but DSMO toxicity results in animals living to median survival of 6.7 days, and the drug proposed brings the survival to 16 days.

Referee #2 (General Remarks):

1. It is unclear why the authors then chose to go in vivo with the 20 mg/kg dosage versus the 5 mg/kg that showed better increases.

The data presented in Fig. 5A were based on preliminary data with a small number of animals. Although a stronger increase with lower doses of LDN 76070 was observed, we chose to proceed with the higher doses in order to provide a greater chance of detecting in vivo efficacy. This has been addressed in the text.

2. Concerning is the DMSO toxicity, and then the drug increasing survival to what normal SMA delta 7 animals live to. It is suggested that the authors do not hide this fact, and should put untreated animals on the survival curve as well as it is unclear whether the drug does anything to

improve survival versus diminish a DMSO toxicity effect.

There is some variability in the survival of SMNΔ7 mice from colony to colony. The "normal" lifespan for the SMNΔ7 animals our experience is 10-12 days. We included untreated mice in all our experiments. Because we considered the DMSO controls as the true comparison, we did not include data from the untreated controls. No untreated mice survive to 15 days in our experience. These data are included in the revised Figure 6 and in the text.

In our colony, untreated animals had an average lifespan of 11.5 days and illustrated lower peak weight gain and diminished gross motor function when compared to LDN-76070 treated animals. The survival increases between untreated and LDN-76070 and between DMSO and LDN-76070 were 140% and 48%, respectively. It is important to note that the differences are statistically significant for both comparisons. The 48% increase in survival is as good or better than most published reports for novel pre-clinical small molecules for treating SMA. We suggest that our treatment not only corrects the SMA phenotype but also allows the SMNΔ7 neonates to overcome the potential sensitivity to DMSO observed with the DMSO treatment alone. This is now clarified in the text.

3. Has the authors considered treating the dams instead of new-born pups? That may be able to circumvent the DMSO toxicity in a new-born pup.

We considered treating the dams. This remains a possibility for future studies. However we do not plan such studies for LDN-76070 or LDN-75654 as we continue development of our compounds.

4. I would like to see a dose response of DMSO toxicity, and an attempt to capture increase in life span with the drug when animals live a normal lifespan.

See discussion of dosing and DMSO above. Comparison of drug treated animals with untreated animals illustrates a statistically significant increase in lifespan for treated animals and addresses this reviewer's concern that the drugs are not effective and dispelling any ambiguity on the interpretation and significance of the results.

5. Another way to address this is to perform electrophysiology in the mice. Are the authors truly correcting the SMA electrophysiological phenotype?

We have not chosen to address the electrophysiology in these mice. We believe the time to right assay provides a sufficient measure of gross motor function for studies in early pre-clinical leads.

6. There is excitement by this reviewer on the screen and potential here, but in present state; the authors appear to be overly optimistic on the drug candidates chosen with significant hurdles that are only briefly touched.

The proof-of-principle data clearly indicate these two series as new and promising leads for SMA therapeutics with both in vitro and in vivo activity. This further validates the novel "hybrid gene" discovery platform that was used to identify our hits. Lead optimization and chemical and pharmacokinetic analysis are underway and are beyond the scope of this manuscript.

Referee #3 (Comments on Novelty/Model System):

My concern is described as major points in the referee comment. The new compound identified in this study has low solubility (requires DMSO); therefore, there is still lot to do to proceed to the clinical application.

I recommend this article can be published as a short report (when appropriately revised), since the study mainly focused to develop new therapeutic compounds for SMA with limited elucidation of disease mechanism.

Referee #3 (General Remarks):

Major points:

1. To date, many studies (more than 20 publications) have identified the compounds to increase the level of SMN2 proteins. In light of these, the novelty and the advantage of new compound should be clearly addressed. The impact of prolonged lifespan in this study can be discussed in comparison with other preclinical studies.

This has been addressed in the text. See the last 3 pages of the discussion.

2. In the discussion, authors mentioned that the compound LDN-76070 showed low solubility to the water (requires DMSO to solubilize). In addition, the penetrance to the central nervous system (through the brain-blood barrier (BBB)) of this compound may be dependent on the immature BBB of the new-born animal. Still, a substantial effort seems to be required to overcome these problems in order to proceed to the clinical application to these compounds.

These are exciting in vitro and in vivo proof-of-principle data for two new series of compounds. A medicinal chemistry program is underway for both scaffolds. We agree that there is still much to do before we could proceed to human trials.

Minor points:

1. page 4, line 13: Italic font should be corrected.

This has been corrected in the text.

2. p9, lines 15, 16. Fold changes of SMN increase, 1.9 fold (1.1 uM of LDN-75654), 1.8 fold (370nM of LDN-76070) do not match with the value provided in the figure 3A.

The data in the figure are from a single experiment. The text describes the average increase from multiple experiments. This has been addressed in the text.

3. p10, line 8. The data provided in fig 3C does not seem to show that the dose-dependent effect of the compounds on the number of gems.

This has been corrected and addressed in the text.

4. Page 17-19: discussion regarding development of LDN-76070 seems too long. It can be shortened.

This has been address in the text.

5. Figure 3B: Scale bar should be included.

We have included scale bars in Fig. 3B.

6. Figure 5A, B: Although HET seems heterozygous mice, it should be noted in the legend. LDN-75654 (18mg/kgG): "G" should be eliminated.

This has been corrected in the text.

7. Figure 5B: what is IP90?

This is a previously utilized house keeping protein used as a loading control and has been described in the text and in the legend for Fig. 5.

2nd Editorial Decision 03 May 2013

Thank you for the submission of your manuscript to EMBO Molecular Medicine. We have now heard back from Reviewer 2, whom we asked to re-evaluate your manuscript.

You will see that Reviewer 2 is still concerned with the effects of DMSO toxicity and is not satisfied that the issues raised were adequately addressed. S/he notes that dosing of the drug at higher concentrations to deliver at DMSO concentrations that do not affect animal survival so much was not carried out. Reviewer 2 is also concerned about how weight gain data are presented in Fig. 6. I concur with this Reviewer and indeed in my initial decision letter I had stressed that this fundamental issue required thorough and convincing action to dispel any ambiguity on the interpretation and exact significance of the results.

It is EMBO Molecular Medicine policy to allow a single round of revision only but I am prepared to allow you to submit a re-revised version that must include a complete response to the Reviewer including the required experimental verification. Acceptance or rejection of the manuscript will

depend on the completeness of your responses included in the next, final version of the manuscript.

As you know, EMBO Molecular Medicine has a "scooping protection" policy, whereby similar findings that are published by others during review or revision are not a criterion for rejection. However, I do ask you to get in touch with us after three months if you have not completed your revision, to update us on the status. Please also contact us as soon as possible if similar work is published elsewhere.

I look forward to seeing a revised form of your manuscript as soon as possible.

***** Reviewer's comments *****

Referee #2 (General Remarks):

The authors have only partly addressed the concerns in the review, and that is related to slanting the data for comparison between the drug vs. DMSO related toxicity. It seems that the authors should attempt to perform some dosing of their compound at a higher concentration (2x, 5x) in order to deliver the drug at a concentration that does not result in animals dying so early. If this is true, then their results (rescue) may be better! For example in Avila et al J Clin. Invest. 2007, the authors used DMSO to disolve the TSA and did not see related toxicity.
Also, I'm not sure how to evlaute the increase of weight gain in animals in fig 6b and 6c, it appears that the peak of animals with DMSO only results in a near similar increase of weight to the compound treated group (black vs. green lines) Do the authors mean, peak weight at the time of median survival? if so, that is a bit misleading.

2nd Revision - authors' response 20 March 2013

Referee #2 (General Remarks):

The authors have only partly addressed the concerns in the review, and that is related to slanting the data for comparison between the drug vs. DMSO related toxicity. It seems that the authors should attempt to perform some dosing of their compound at a higher concentration (2x, 5x) in order to deliver the drug at a concentration that does not result in animals dying so early. If this is true, then their results (rescue) may be better! For example in Avila et al J Clin. Invest. 2007, the authors used DMSO to dissolve the TSA and did not see related toxicity.

There remains to be concern about the toxicity of DMSO and its effect comparative to untreated animals. It is standard to compare drug treatment to the vehicle control, in this case DMSO. Reviewer 2 seems to focus on the untreated mice for comparison. Our data show statistically significant extension of life and motor capabilities with LDN-76070 in comparison to both DMSO vehicle and untreated animals. Reviewer 2 also appears to ignore the physical motor data in the “time to right assay”. Clearly there are significant improvements with LDN 76070 over both DMSO and untreated animals.

Reviewer 2 implies that DMSO is an inert vehicle for injection. We disagree. The low aqueous solubility of LDN-76070 required us to use DMSO. Reviewer 2 referenced *Avila et al J Clin. Invest. 2007*. The animals in that paper were injected with 2.5 uL of compound in DMSO (equivalent to the LD_{50} for healthy adult mice). These authors did not provide the median lifespan for untreated SMA animals so we do not know the effects of DMSO. In this publication, mice also had a longer median lifespan with DMSO treatment than our untreated animals. Variations such as this have been previously observed with different colonies of the SMNΔ7 animals.

We reiterate that the increase in survival observed with LDN-76070 is as good as or better than any published small molecules for the treatment of SMA, even in comparison to *Avila et al*. Furthermore, I have included additional data, for a point of reference and not to be included in the manuscript, in which we treated animals every other day with LDN-76070 to diminish the cumulative amount of DMSO and allow time for recovery from the intraperitoneal injections.

Consistent with the data in our manuscript, there were significant increases in survival and improvement in gross motor function.

Weight Gain for Compound 76070 Treated Mice

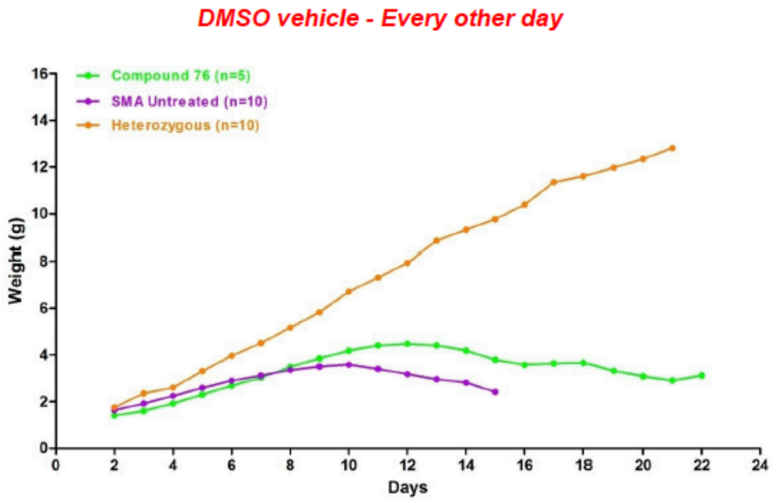

Survival proportions for Compound 76070 Treated Mice

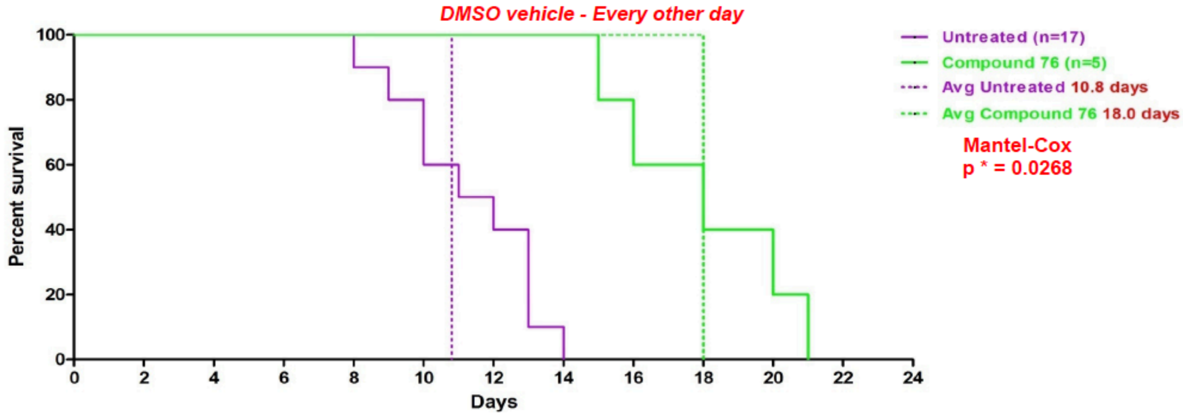

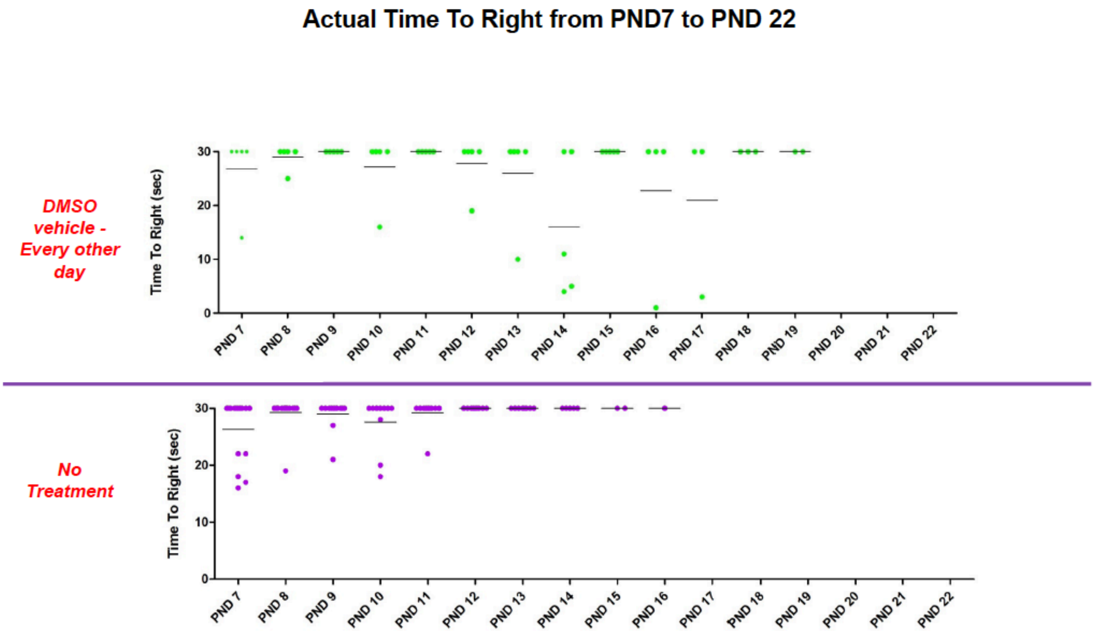

Also, I'm not sure how to evaluate the increase of weight gain in animals in fig 6b and 6c, it appears that the peak of animals with DMSO only results in a near similar increase of weight to the compound treated group (black vs. green lines) Do the authors mean, peak weight at the time of median survival? if so, that is a bit misleading.

We apologize for confusion about figures 6b,c. There was no attempt to "mislead". Figure 6b presents the average peak weight gain of all animals including those that died on days 3-6. On average these animals were smaller when they died and caused the average peak weight gain to be lower. Figure 6c presents the average weights for surviving animals on each specified day. Since the sicker, smaller animals (6/10) all died on or before day 6, this causes an apparent increase in average weight on days 7 through 10. In reality, remaining animals were always larger and the average weight on each day is not impacted by the dead mice. We would state this clearly in the re-submission and could provide all the weights in a supplement, but do not believe this is warranted as survival and motor skills are much more critical.

To address this the following changes made in manuscript:

pg 15 Line 8-10 "The weights of the animals were recorded each day and the average weight gain from birth to peak for all animals in each tested group was calculated (Fig. S2)."

pg 15 Line 14-20 "We also calculated the daily average weights for each of the surviving animals, including the sicker, smaller animals that died prematurely (on or before day 6). Since the median survival of the DMSO treated animals was 6 days, the apparent increase in their average weight on days 7 through 10 was the result of the limited number of animals remaining; each of these had slightly higher weights (Fig. 6C and Fig. S2). However, the animals treated with LDN-76070 still reached a higher average peak weight of 5.05 ±0.9 g and had substantially longer life span compared to either of the control groups (Fig. 6C and Fig. S2)."

pg 16 Line 6-7 "Collectively, the data show statistically significant extension of life and motor capabilities with LDN-76070 treatment in comparison to DMSO and untreated animals."

pg 40 Line 1 "Average percent weight gained from peak to birth"

pg 40 Line 4 " surviving members"

Addition of Supplemental Figure S2

pg 40-41 lines 21-23, 1-4 "Figure S2. Weights of treated and control animals. Weights of all animals were recorded each day. The average peak weight and time to reach peak weight were determined for each animal. Increase in weight from birth to peak was also determined for each animal and the average fold increase (FI) and percent increase (% Incr.) in weight for each group

was calculated for Fig. 6B. The weights for all surviving animals on each day were used to determine the average weight of the surviving population on that particular day. These data were used to generate Fig. 6C. The table summarizes the results for each group of animals."

3rd Editorial Decision 25 March 2013

Thank you for the submission of your revised manuscript to EMBO Molecular Medicine. I am pleased to inform you that we will be able to accept your manuscript pending the following final technical amendments:

1) The description of all reported data that includes statistical testing must state the name of the statistical test used to generate error bars and P values, the number (n) of independent experiments underlying each data point (not replicate measures of one sample), and the actual P value for each test (not merely 'significant' or 'P < 0.05'). Please make sure that this if fully complied with.

3) There is space at the end of each article to list relevant web links for further consultation by our readers ("For more information"). Could you identify some relevant ones and provide such information as well? Some examples are patient associations, relevant databases, OMIM/proteins/genes links, author's websites, etc... (this is not compulsory but might apply in your case)

Please submit your revised manuscript within two weeks. Needless to say, the sooner we receive it the sooner I will be able to formally accept your manuscript.

I look forward to reading a new revised version of your manuscript as soon as possible.

© EMBO
