# Supplementary material for: Enhancement of SMN protein levels in a mouse model of spinal muscular atrophy using novel drug-like compounds
Source: EMBO Mol Med. 2013 Jun 5;5(7):1035–50. doi: 10.1002/emmm.201202305 (PMC3721476; doi:10.1002/emmm.201202305)
Supplement: Supplementary file 2 [file emmm0005-1035-SD2.pdf]

Supporting Information

Enhancement of SMN protein levels in a mouse model of spinal muscular atrophy using novel drug-like compounds.

Jonathan J. Cherry, Erkan Y. Osman, Matthew C. Evans, Sungwoon Choi, Xuechao Xing, Gregory D. Cuny, Marcie A. Glicksman, Christian L. Lorson, and Elliot J. Androphy

Supporting Information Figures

Figure S1: **Schematic of reporter mini-genes.**

Figure S2: **Weights of treated and control animals**

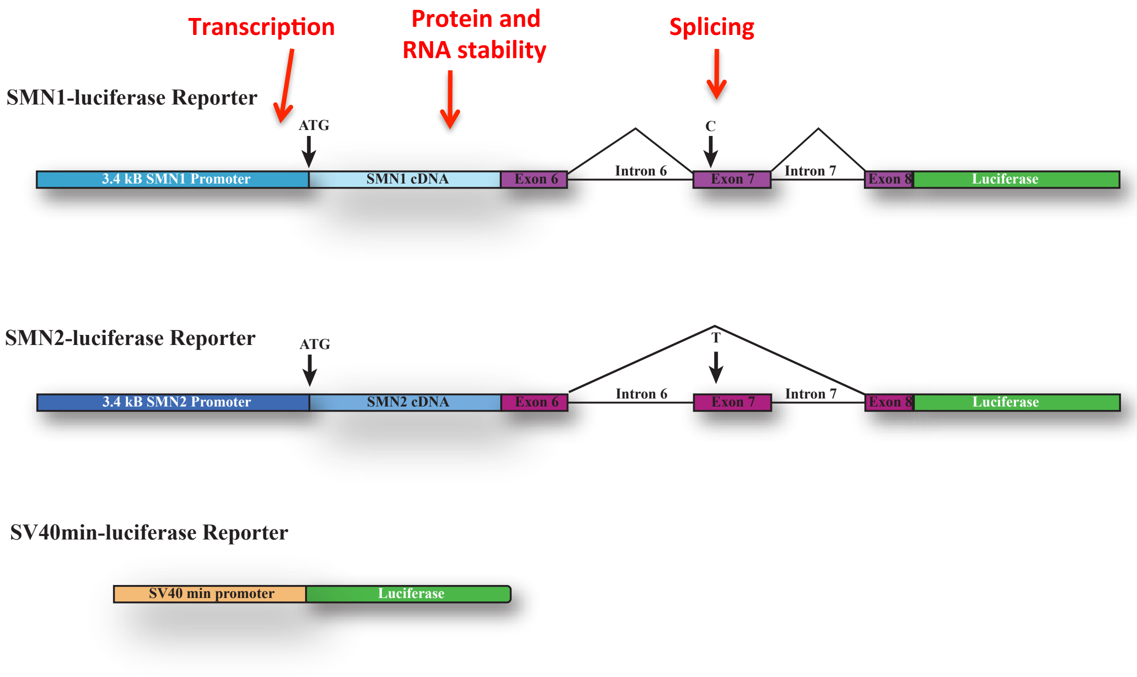

Figure S1. Schematic of reporter mini-genes. The SMN-luciferase reporters are each driven by their respective promoters (dark blue), contain the cDNA for exons 1-5 (light blue), and the genomic splicing cassette for exons 6-8 (purple) (Cherry et al, 2012). A frameshift mutation was engineered into the stop codon in exon 7. This results in a change in the coding sequence that allows for the expression of luciferase (green) only when exon 7 is included. Transcripts that undergo alternative splicing and exclude exon 7 will not express luciferase. The nucleotide variation at position 6 in exon 7 (C or T) is noted for each construct. The predominant splicing pattern for each construct is also displayed. For SMN 1 > 85% of the transcripts contain exon 7, while only 10-15% of SMN2 transcripts contain exon 7. These reporters are able to detect changes in SMN transcription, SMN protein or mRNA stability, and inclusion of SMN exon 7. The *SV40min-luciferase* reporter was used as a specificity control. This construct contains the luciferase cDNA under the control of the SV40 minimal promoter (yellow). It measures non-specific changes in transcription, luciferase stability, and luciferase enzymatic activity.

DAY	Compound LDN 76 (n=19) weight in grams																			Average per day (g)	
1	1.54	1.63	1.31	1.73	1.28	1.72	1.49	1.34	1.44	1.62	1.59	1.33	1.21	1.41	0.98	1.43	1.23	1.84	1.59	**1.46**	
2	1.89	1.92	1.66	2.01	1.67	2.03	1.81	1.67	1.71	1.91	1.9	1.77	1.45	1.75	1.16	1.74	1.51	2.17	1.88	**1.77**	
3	2.24	2.38	1.93	2.16	1.85	2.39	2.09	1.98	2.01	2.29	2.25	2.19	2.01	2.46	1.61	2.18	2.04	2.61	2.37	**2.16**	
4	2.88	2.99	2.32	2.54	2.35	3.07	2.52	2.49	2.51	2.85	2.86	2.71	2.36	2.77	1.91	2.51	2.31	3.02	2.67	**2.61**	
5	3.29	3.46	2.64	2.98	2.74	3.61	3.09	2.82	3.01	3.46	3.41	2.95	2.68	3.19	2.18	2.9	2.6	3.49	3.17	**3.04**	
6	3.69	3.85	2.93	3.39	3.21	4.21	3.56	3.06	3.36	3.94	3.86	3.28	2.95	3.72	2.53	3.37	2.96	4.01	3.91	**3.46**	
7	4.13	4.26	**2.95**	3.87	3.73	4.71	4.01	3.46	3.69	4.54	4.48	3.86	3.15	4.38	2.99	3.81	3.29	4.32	4.55	**3.90**	
8	4.51	4.57	2.63	4.4	4.2	5.42	4.32	3.78	4.02	5.13	5.07	4.32	3.98	4.8	3.3	4.11	3.53	4.83	5.23	**4.32**	
9	4.49	4.75		4.68	4.46	5.61	4.38	3.78	4.13	5.64	5.58	4.73	4.41	5.45	3.62	4.61	3.74	5.17	**6.12**	**4.74**	
10	**4.59**	4.8		5.06	4.52	**6.23**	4.72	**3.96**	**4.41**	5.99	5.98	**4.81**	4.67	5.73	4.08	**4.98**	**3.88**	**5.31**	4.91	**4.92**	
11	4.37	**4.85**		5.68	4.73	6.11	**4.92**	3.79	3.87	**6.11**	**6.05**	4.53	**5.15**	**5.88**	4.42	4.67	3.72	4.87	4.64	**4.91**	
12	4.22	4.53		**6.18**	**4.74**	5.64	4.9	3.54		5.94	5.62		4.91	5.49	**4.31**	4.34	3.62	4.58	4.29	**4.80**	
13	3.91	4.27		5.91	4.54	5.48	4.55	3.37		5.31	5.12		4.67	5.07	4.29	3.98	3.33	4.23		**4.54**	
14	3.69	3.89		5.42	4.21	5.22	4.18			4.92	4.85		4.29	4.62	3.98	3.67	3.08	3.92		**4.28**	
15	3.32	3.59		5.07	3.92	4.9	3.91			4.56	4.54		4.02	4.29	3.74	3.39	2.85	3.65		**3.98**	
16	3.11	3.23		4.72	3.61	4.58	3.69			4.24	4.26		3.81	3.95	3.46	3.23	2.76	3.48		**3.72**	
17				4.42	3.52	4.23	3.32			3.92	3.99		3.78	3.82	3.27	2.79	2.52	3.21		**3.57**	
18				4.22	3.21	4.02	3.12			3.69	3.66		3.36	3.38	2.91			2.98		**3.46**	
19				3.97	2.85	3.68				3.45	3.31			3.03						**3.38**	
20						3.38								2.78						**3.08**	
21																				**Average**	
	2.98	2.98	2.25	3.57	3.70	3.62	3.30	2.96	3.06	3.77	3.81	3.62	4.26	4.17	4.40	3.48	3.15	2.89	3.85	**3.46**	**FI**
	198.1	197.5	125.2	257.2	270.3	262.2	230.2	195.5	206.3	277.2	280.5	261.7	325.6	317.0	339.8	248.3	215.4	188.6	284.9	**246.39**	**% Incr.**

DAY	DMSO (n=10) weight in grams									Average per day (g)	
1	1.32	1.01	1.15	1.09	1.52	1.56	1.48	1.49	1.11	1.71	**1.34**	
2	1.43	1.12	1.23	1.21	1.74	1.78	1.65	1.71	1.37	2.02	**1.53**	
3	1.92	1.33	1.66	1.48	2.01	1.94	1.87	1.75	**1.31**	**2.31**	**1.76**	
4	2.32	1.98	2.14	**1.58**	2.41	2.32	2.15	1.83			**2.09**	
5	2.82	**2.3**	2.58	1.32	2.81	2.72	2.48	1.89			**2.37**	
6	3.37		**2.86**		3.29	3.08	2.81	**1.96**			**2.90**	
7	3.72				3.78	3.51	3.17				**3.55**	
8	4.21				**4.12**	3.87	3.38				**3.90**	
9	4.48				3.61	4.39	3.61				**4.02**	
10	**4.61**					**4.47**	**4.02**				**4.37**	
											**Average**	
	3.49	2.28	2.49	1.45	2.71	2.87	2.72	1.32	1.18	1.35	**2.18**	**FI**
	249.2	127.7	148.7	45.0	171.1	186.5	171.6	31.5	18.0	35.1	**118.4**	**% Incr.**

DAY	Untreated (n=10) weight in grams									Average per day (g)	
1	1.54	1.21	1.34	1.51	1.32	1.3	1.19	1.22	1.56	1.67	**1.39**	
2	1.71	1.59	1.62	1.72	1.62	1.68	1.41	1.43	1.72	1.98	**1.65**	
3	1.97	1.82	1.89	2.01	1.95	1.93	1.64	1.78	1.87	2.08	**1.89**	
4	2.18	2.07	2.11	2.29	2.18	1.96	1.85	2.22	2.23	2.67	**2.18**	
5	2.55	2.42	2.39	2.5	2.37	2.24	1.95	2.68	2.6	3.11	**2.48**	
6	2.82	2.68	2.54	2.76	2.61	2.75	**2.02**	3.01	2.87	3.39	**2.75**	
7	3.11	2.91	2.88	2.92	2.87	2.76	2.02	3.19	3.08	3.67	**2.94**	
8	**3.34**	3.22	3.01	3.17	**2.96**	3.02	2.01	3.42	3.23	4.09	**3.15**	
9	3.25	**3.41**	**3.18**	**3.26**	2.84	3.18	1.98	3.59	3.44	4.23	**3.24**	
10		3.17	3.02	2.99	2.58	3.22	1.86	**3.91**	**3.52**	**4.46**	**3.19**	
11		2.98	2.83	2.61	2.49	**3.28**	1.71	3.72	3.12	3.97	**2.97**	
12				2.54		3.15		3.61	2.88	3.78	**3.19**	
13						2.92		3.35		3.12	**3.13**	
14						2.62		3.15		2.89	**2.89**	
											**Average**	
	2.17	2.82	2.37	2.16	2.24	2.52	1.70	3.20	2.26	2.67	**2.41**	**FI**
	116.9	181.8	137.3	115.9	124.2	152.3	69.7	220.5	125.6	167.1	**141.1**	**% Incr.**

	Time to peak	Average weight at birth (g)	Average weight at peak (g)	Average weight increase (g)	average fold increase	average % increase
**LDN-76070**	10.4 days	1.45	5.03	3.58	3.46	246.39
**DMSO**	6.5 days	1.34	2.9	1.56	2.18	118.45
**Untreated**	9 days	1.38	3.3	1.92	2.41	141.14

Figure S2. Weights of treated and control animals. Weights of all animals were recorded each day. The average peak weight and time to reach peak weight were determined for each animal. Increase in weight from birth to peak was also determined for each animal and the average fold increase (FI) and percent increase (% Incr.) in weight for each group was calculated for Fig. 6B. The weights for all surviving animals on each day were used to determine the average weight of the surviving population on that particular day. These data were used to generate Fig. 6C. The table summarizes the results for each group of animals.
